# Expert-guided protein language models enable accurate and blazingly fast fitness prediction

**DOI:** 10.1093/bioinformatics/btae621

**Published:** 2024-11-22

**Authors:** Céline Marquet, Julius Schlensok, Marina Abakarova, Burkhard Rost, Elodie Laine

**Affiliations:** School of Computation, Information, and Technology, Department of Informatics, Bioinformatics and Computational Biology, Technical University of Munich, Garching/Munich 85748, Germany; School of Computation, Information, and Technology, Department of Informatics, Bioinformatics and Computational Biology, Technical University of Munich, Garching/Munich 85748, Germany; Laboratory of Computational and Quantitative Biology, UMR 7238, Sorbonne Université, CNRS, IBPS, Paris 75005, France; UMR U1284, Universite Paris Cité, INSERM, Paris 75004, France; School of Computation, Information, and Technology, Department of Informatics, Bioinformatics and Computational Biology, Technical University of Munich, Garching/Munich 85748, Germany; School of Life Sciences Weihenstephan, Technical University of Munich, Freising, Germany; Laboratory of Computational and Quantitative Biology, UMR 7238, Sorbonne Université, CNRS, IBPS, Paris 75005, France; Institut Universitaire de France, Paris 75005, France

## Abstract

**Motivation:**

Exhaustive experimental annotation of the effect of all known protein variants remains daunting and expensive, stressing the need for scalable effect predictions. We introduce VespaG, a blazingly fast missense amino acid variant effect predictor, leveraging protein language model (pLM) embeddings as input to a minimal deep learning model.

**Results:**

To overcome the sparsity of experimental training data, we created a dataset of 39 million single amino acid variants from the human proteome applying the multiple sequence alignment-based effect predictor GEMME as a pseudo standard-of-truth. This setup increases interpretability compared to the baseline pLM and is easily retrainable with novel or updated pLMs. Assessed against the ProteinGym benchmark (217 multiplex assays of variant effect—MAVE—with 2.5 million variants), VespaG achieved a mean Spearman correlation of 0.48 ± 0.02, matching top-performing methods evaluated on the same data. VespaG has the advantage of being orders of magnitude faster, predicting all mutational landscapes of all proteins in proteomes such as *Homo sapiens* or *Drosophila melanogaster* in under 30 min on a consumer laptop (12-core CPU, 16 GB RAM).

**Availability and implementation:**

VespaG is available freely at https://github.com/jschlensok/vespag. The associated training data and predictions are available at https://doi.org/10.5281/zenodo.11085958.

## 1 Introduction

Proteins are the essential building blocks of life, fulfilling a wide range of vital roles within cells and organisms. Hence, understanding the effect of variations such as point mutations on protein stability and function is crucial for comprehending disease mechanisms (Murray *et al.* 2017) and modulating their activities through engineering. Multiplexed assays of variant effect (MAVEs), in particular deep mutational scans (DMS) (Fowler and Fields 2014), have enabled the quantification of mutational outcomes on a much larger scale than ever before. They allow for an in-depth characterization of protein mutational landscapes by assessing the impact of virtually all possible single amino acid substitutions. Nevertheless, conducting experimental assays for entire proteomes remains elusive (Atlas of Variant Effects Alliance, https://www.varianteffect.org).

Leveraging the power of computational models can help to gain insights into the functional consequences of protein variants and to prioritize them for further experimental validation. However, the sparseness of annotations challenges the development of such models. While many supervised machine learning (ML) methods have proven accurate (Adzhubei *et al.* 2013, Hecht *et al.* 2015, Gray *et al.* 2018), they are inherently biased toward the limited number of proteins characterized by MAVEs or having annotated disease-associated variants (Livesey and Marsh 2023). As a result, different methods tend to correlate highly for the tiny subset of experimental data, while their predictions for, e.g. all possible mutations in the human proteome correlate very poorly (Hecht *et al.* 2013, Mahlich *et al.* 2017). Prediction methods are also sensitive to the noise and uncertainty in these data. MAVE annotations, for instance, may vary substantially across experiments, even when measuring the same phenotype for the same protein (Reeb *et al.* 2020). These difficulties have stimulated a growing interest in unsupervised or weakly supervised methods predicting variant effects by only exploiting information from protein sequences observed in nature (Ng and Henikoff 2003).

Among the best-performing unsupervised methods, GEMME explicitly models the evolutionary history of protein sequences (Laine *et al.* 2019, Notin *et al.* 2023). Starting from a multiple sequence alignment (MSA), it determines how protein sites are segregated along the topology of phylogenetic trees to quantify the sensitivity of each site to mutations and the number of changes required to accommodate a substitution. It relies on only a few biologically meaningful parameters and is robust to low variability in the input MSA. GEMME proved instrumental for investigating the interplay between protein stability and function, and elucidating disease mechanisms (Abildgaard *et al.* 2023, Cagiada *et al.* 2023, Gersing *et al.* 2023, Tiemann *et al.* 2023, Tsuboyama *et al.* 2023). Combining GEMME with a fast MSA generation algorithm allows for producing proteome-wide substitution score matrices within a few days (Abakarova *et al.* 2023).

Other methods rely on protein language models (pLMs) pretrained over large databases of raw sequences (Elnaggar *et al.* 2022, Lin *et al.* 2023). The log-odds ratios computed from the masked marginal probabilities can already provide highly accurate estimates of mutational effects (Meier *et al.* 2021, Livesey and Marsh 2023). Nevertheless, the quality of the protein sequence representations learned by foundation pLMs is highly variable, and especially poor for viral proteins (Elnaggar *et al.* 2022, Lin *et al.* 2023, Notin *et al.* 2023, The UniProt Consortium 2023, Ding and Steinhardt 2024). While pLM performance can be further boosted through incorporating information about evolutionary conservation, population genetic polymorphism and 3D structures (Meier *et al.* 2021, Marquet *et al.* 2022, Nijkamp *et al.* 2023, Notin *et al.* 2022a, Cheng *et al.* 2023, Truong and Bepler 2023, Su *et al.* 2024), the computational cost of zero-shot inference over full-length proteins remains high.

**Figure 1.**
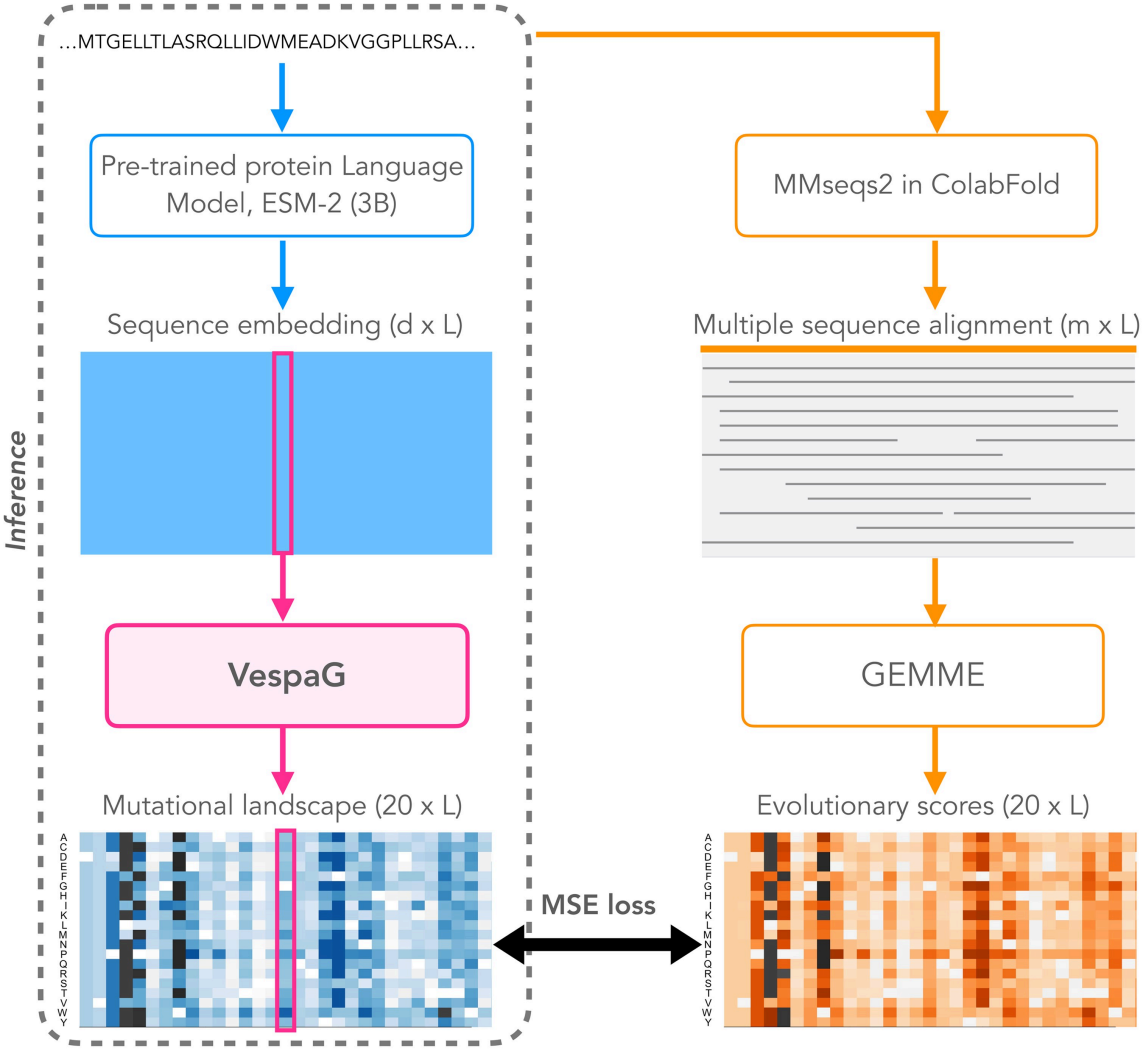
Outline for VespaG’s expert-guided approach. VespaG takes as sole input a *d* = 2560-dimensional vector representation of a wild-type residue in a protein computed by the pretrained protein language model (pLM) ESM-2 with 3 billion parameters (Lin *et al.* 2023), and outputs a 20-dimensional vector of predicted mutational outcome estimates. The training loss measures the mean squared error between the predicted estimates and the evolutionary scores computed by GEMME (Laine *et al.* 2019). We generate millions of training samples through the MMseqs2-based ColabFold protocol for searching and aligning sequences (Steinegger and Söding 2017, Mirdita *et al.* 2022, Abakarova *et al.* 2023). We do not use alignments at inference time (dotted rectangle). VespaG’s framework can be adapted to any pretrained pLM.

Here, we optimized prediction speed by circumventing the computationally expensive masked token reconstruction task and directly mapping pLM embeddings to complete mutational landscapes using the evolutionary-informed model GEMME as a teacher (Fig. 1). To this end, we trained a comparatively shallow (660k free parameters) neural network on top of a pretrained pLM without computing log-odds ratios to learn GEMME predictions. Our strategy overcomes the bottleneck of sparsely annotated experimental training data. Moreover, it avoids the noise and inconsistencies of the experimental assays. We implemented our approach as a lean tool for fast *V*ariant *E*ffect *S*core *P*rediction without *A*lignments enabled by *G*EMME (VespaG). We assessed prediction performance against over 3 million (M) missense variants across diverse protein families. VespaG performed on par with state-of-the-art (SOTA) methods and in some cases, the student even surpassed the teacher GEMME. As we circumvent the need to compute log-odds ratios of substitution probabilities, VespaG enables proteome-wide predictions in less than a half hour on a standard consumer laptop. We also demonstrated VespaG to generalize across organisms and protein families.

## 2 Materials and methods

### 2.1 Comparison to state-of-the-art methods

We compared VespaG to seven SOTA predictors, namely *GEMME* (Laine *et al.* 2019) as it is (1) tied for the best performing method on ProteinGym (Notin *et al.* 2023, https://github.com/OATML-Markslab/ProteinGym), (2) a purely MSA-based method not using machine learning, and (3) was used to annotate VespaG’s training data; zero-shot log-odds by the pLMs *ESM-2* (Lin *et al.* 2023), the sequence-only pLM used as input to VespaG and *SaProt* (Su *et al.* 2024), a top-ranked pLM in ProteinGym which takes structure and sequence as input; *TranceptEVE L* (Notin *et al.* 2022b) as it is the best performing method on ProteinGym next to GEMME and SaProt, and because it is a hybrid model, making use of both MSAs and pLM embeddings as input, combining the previously developed autoregressive Tranception (Notin *et al.* 2022a) with the Bayesian variational autoencoder EVE (Frazer *et al.* 2021); *PoET* (Truong and Bepler 2023), a recently developed autoregressive generative method modeling protein families as sequences-of-sequences and slightly outperforming other methods against the first version of the ProteinGym set; *AlphaMissense* (Cheng *et al.* 2023), also recently introduced and building up on the protein structure predictor AlphaFold (Jumper *et al.* 2021) by incorporating population frequency data; and *VESPA* (Marquet *et al.* 2022), which predicts per-residue conservation scores and combines them with per-mutation protein-dependent log-odds scores and per-mutation protein-independent substitution scores, as it is currently the best purely sequence pLM-based method in ProteinGym. We mainly relied on the Spearman rank correlation coefficient to assess predictive performance. PoET and AlphaMissense were evaluated on the first ProteinGym iteration but are not included in the updated benchmark (see Supplementary Methods for details on producing or retrieving the predictions).

### 2.2 Method development

#### 2.2.1 Datasets

To generate training data, we constructed a main set based on the *Homo sapiens* proteome and additional sets representing diverse origins, namely *Drosophila melanogaster*, *Escherichia coli*, as well as all viruses (Supplementary Table S1). Each training dataset was curated following the same process of first downloading the UniProt (The UniProt Consortium 2023) reference proteome(s) with one protein sequence per gene and removing any proteins of less than 25 or more than 1024 residues. We redundancy reduced the training data in two steps, firstly against the test data to prevent data leakage and secondly against themselves to reduce the number of training samples–see Supplementary Methods for details. We generated training and validation sets using a random 80/20 split. To circumvent the need for a large, comprehensive set of experimental variant effect annotations, we employed the established method GEMME following the protocol introduced in (Abakarova *et al.* 2023). Specifically, for each protein from the training set, we retrieved and aligned a set of homologous sequences with the MMseqs2-based multiple sequence alignment (MSA) generation strategy implemented in ColabFold (Mirdita *et al.* 2022). We then used the generated MSA as input for GEMME. GEMME outputs a complete substitution matrix of dimension *L* × 20, with *L* being the length (in residue) of the input query protein sequence. GEMME scores range from −10 to 2. Drawing from our previous findings (Abakarova *et al.* 2023), we flagged the mutational landscapes derived from fewer than a couple hundred homologous sequences as lowly confident.

Additionally, we compared VespaG against SOTA methods on the two test sets *ProteinGym* (with nine subsets) and *StabilityDeNovo146*. The substitution benchmark *ProteinGym* (Notin *et al.* 2023) comprised 217 DMS from 187 unique proteins with diverse lengths (37–3423 residues with a median of 245), protein families (e.g., polymerases, tumor suppressors, kinases, and transcription factors), sizes, functions (e.g. drug resistance, ligand binding, viral replication, and thermostability), and taxa, totalling about 696k single missense variants and 1.76M multiple missense mutations from 69 of the 217 proteins (Supplementary Fig. S3). The first iteration of the benchmark, which we also considered, contained 87 DMS from 73 unique proteins (72 − 3423 residues with a median of 379), totaling about 1.5M variants with mostly single and, for 11 proteins, multiple missense mutations (Supplementary Fig. S4; see Supplementary Methods for more details). To additionally assess the predictors on *de novo* domains, we compiled the test set *StabilityDeNovo146* from the most comprehensive available dataset assessing how amino acid substitutions affect thermodynamic folding stability (Tsuboyama *et al.* 2023). *StabilityDeNovo146* comprises 123k variants across 146 proteins designed using TrRosetta (Yang *et al.* 2020) with the hallucination protocol described in (Anishchenko *et al.* 2021, Norn *et al.* 2021) or the blueprint-based approach described in (Huang *et al.* 2011, Kim *et al.* 2022). We selected all mutations with tabulated free energy changes (ΔΔ*G*) annotations, discarded all deletions, insertions, and wild-type sequences, and averaged multiple measurements for the same mutation.

#### 2.2.2 Model specifications

All developed models rely solely on embeddings computed from pre-trained pLMs as input. Specifically, we used ProtT5-XL-U50 (Elnaggar *et al.* 2022), an encoder−decoder transformer architecture trained on the Big Fantastic Database (Steinegger and Söding 2017) and fine-tuned on UniRef50, and ESM2-T36-3B-UR50 (Lin *et al.* 2023), a BERT (Devlin *et al.* 2019) style 3-billion-parameter encoder-only transformer architecture trained on all clusters from Uniref50, augmented by sampling sequences from the Uniref90 clusters of the representative chains (excluding artificial sequences). In the following, we refer to these pLMs as ProtT5 and ESM-2, respectively. For both pLMs, we downloaded the encoder weights from HuggingFace (Wolf *et al.* 2020) at https://huggingface.co/docs/transformers/model_doc/esm and extracted the embeddings from the encoder's last hidden layer. These embeddings comprise 1024-dimensional vectors for each residue in a sequence for ProtT5 (Elnaggar *et al.* 2022) and 2560-dimensional vectors for ESM-2 (Lin *et al.* 2023). There is no length restriction for either pLM at inference, so proteins were processed in full. A guide to embedding extraction for ProtT5 and ESM-2 can also be found in our GitHub repository https://github.com/jschlensok/vespag. We used the pre-trained pLMs as is, without fine-tuning their weights, and without combining the embeddings either by concatenating the input or averaging the outputs.

We built the predictors with the following architectures: (1) Linear regression, i.e. a feed-forward neural network (FNN, LeCun *et al.* 2015) without any hidden layer, dubbed *LinReg*; (2) FNN with one dense hidden layer, called *VespaG*; (3) FNN with two hidden layers, called *FNN_2_layer*; (4) Convolutional neural network (CNN, LeCun *et al.* 2015) with one 1-dimensional convolution and two hidden dense layers, referred to as *CNN*; and (5) an ensemble of separately optimized FNN and CNN (with the same architecture as the best stand-alone model for each architecture), with the output being the mean of the two networks. No activation function was used for the output layer. To ease score interpretability, the users can opt for a normalisation of raw VespaG scores to the [0,1] interval where values close to 1 indicate high functional impact, following the Atlas of Variant Effects Alliance guidelines (Livesey *et al.* 2024) (see Supplementary Methods for more details).

## 3 Results

The method introduced in this work, VespaG, is a feed-forward neural network (FNN) with one hidden layer with 256 hidden units solely inputting sequence embeddings from the protein language model (pLM) ESM-2 (Lin *et al.* 2023). We trained VespaG on a set of about 5000 human proteins to learn a mapping between the input pLM embeddings and the evolutionary scores computed by GEMME. The latter served as surrogates for mutational phenotypic outcomes. The proteins used for training represented a non-redundant subset of the human proteome (Methods and Supplementary Table S1).

We initially considered five different architectures for learning from GEMME (“MSE loss” in Fig. 1), including linear regression and convolutional neural networks, and two foundation pLMs, namely ESM-2 (Lin *et al.* 2023) and ProtT5 (Elnaggar *et al.* 2022) (Supplementary Table S2). Performance was similar for all evaluated models (Supplementary Figs S1 and S2), indicating that both pLMs provide robust results under supervision of GEMME regardless of downstream architecture. As we obtained the best performance with a one-hidden-layer FNN and ESM-2 embeddings against the validation set (random 80/20 split, Supplementary Fig. S2), we report test set results for this configuration in the following.

### 3.1 VespaG competitive with state-of-the-art

**Figure 2.**
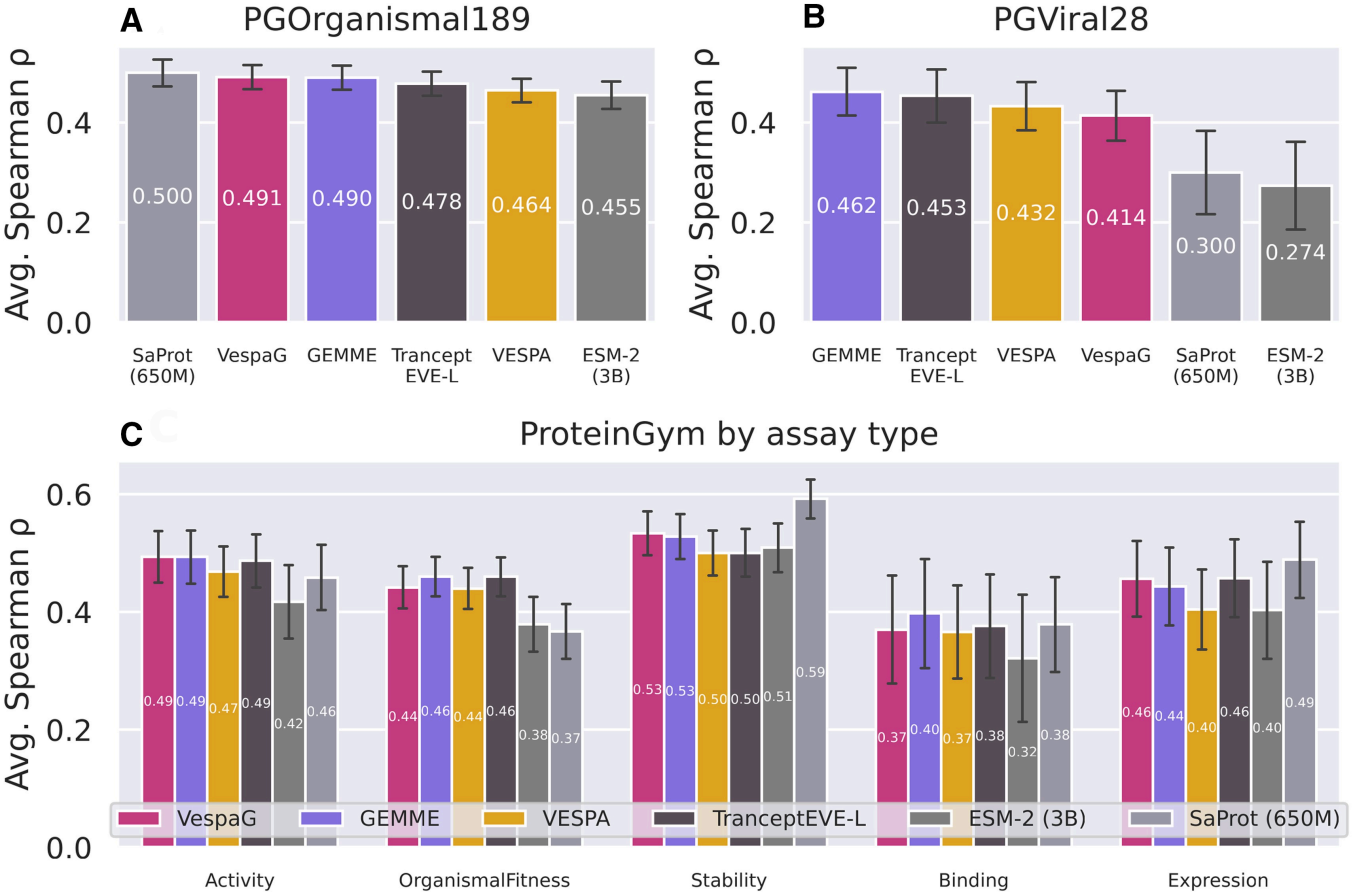
VespaG accuracy on-par with SOTA. Each panel corresponds to a different test set (with partially overlapping proteins between the test sets in C w.r.t. A and B) from the ProteinGym substitution benchmark (Notin *et al.* 2023): (A) *PGOrganismal189*, containing 189 experimental assays for 161 eukaryotic and prokaryotic proteins; (B) *PGViral28*, containing 28 assays for 26 viral proteins; (C) 217 assays in ProteinGym divided into subsets named according to assessed phenotype and number of experiments: *PGActivity43*, *PGBinding13*, *PGExpression18*, *PGFitness77*, and *PGStability66*. For A and B, methods are ordered from best (left) to worst (right), for C we follow a set order. We did not recompute results for TranceptEVE L (Notin *et al.* 2022b), ESM-2 (Lin *et al.* 2023), and SaProt (Su *et al.* 2024), therefore all depicted in shades of gray, and directly extracted the predictions from ProteinGym. The error bars show the 95% confidence interval.

VespaG predicted mutational outcomes with an average overall Spearman correlation coefficient (*ρ*) of 0.480 ± 0.021 (±1.96 standard errors, i.e. 95% confidence interval, CI; Supplementary Methods) against all 217 experimental DMS assays from the ProteinGym substitution benchmark ((Notin *et al.* 2023), June 2024). It performed *on par* with the top methods from the ProteinGym leaderboard, including its teacher GEMME, and substantially better than the zero-shot ESM-2 baseline (Fig. 2, Supplementary Tables S3 and S4).

Most of the assays (189 out of 217) were from proteins from eukaryotic and prokaryotic organisms (Supplementary Fig. S3). On these proteins, VespaG reached an average *ρ* = 0.491 ± 0.024 (Fig. 2A and Supplementary Table S3, *PGOrganismal189*). Its prediction accuracy exceeded all of the following: zero-shot ESM-2 log-odds ratios between the mutant and wild-type amino acids (Δ*ρ* = 0.036, one-tailed paired *t*-test *P*-value < 10^−5^), pLM-based VESPA (Δ*ρ* = 0.027, *P* < 10^−8^) and the ensemble sequence- and MSA-based predictor TranceptEVE L (Δ*ρ* = 0.013, *P* = 0.006). VespaG performed *on par* with its *teacher* GEMME (|Δ*ρ*|< 0.01, *P* > 0.1) and the top-ranked method in ProteinGym (as of June 2024), namely the sequence- and structure-based pLM SaProt (Su *et al.* 2024). Accuracy varied substantially across different experimental DMS assays (Supplementary Fig. S3). Yet, VespaG was stable, in the sense that the distribution of its Δ*ρ* values with respect to the mean *ρ* over the six highlighted methods was very narrow and centered around zero (Supplementary Fig. S5). By contrast, the distributions for the ESM-2 baseline and SaProt were much wider, displaying performance worse than the mean by Δ*ρ* < −0.35 for some assays (Supplementary Figs S5 and S6). Simply put: VespaG appeared to be the most *average* method with the lowest spread between assays (Supplementary Fig. S6). On a subset of *PGOrganismal189*, dubbed *PGOrganismal66*, we could extend the comparison to the SOTA methods AlphaMissense and PoET (predictions not readily available for other data sets). On this subset, VespaG’s predictive performance, with an average *ρ* = 0.484 ± 0.044, outperformed the pLMs SaProt and ESM-2 (Δ*ρ* > 0.27, *P* < 0.007), and was slightly better than GEMME and TranceptEVE-L (Δ*ρ* > 0.07, *P* < 0.04); it was *on par* with PoET (|Δ*ρ*|< 0.01, *P* > 0.1) and comparable to AlphaMissense (Δ*ρ* = −0.021, P ∼ 10^−3^; Supplementary Fig. S7).

The experimental DMS assays represent an unbalanced panel of different phenotypes (Supplementary Fig. S3), namely organismal fitness (*PGFitness77*, highest number of DMS), stability (*PGStability66*), activity (*PGActivity43*), expression (*PGExpression18*), and binding (*PGBinding13*). Balancing the calculation of the average performance according to the phenotypes’ cardinalities yielded *ρ* = 0.459 ± 0.049 for VespaG, outperforming TranceptEVE L, VESPA, SaProt and ESM-2, and bested only by teacher GEMME (Supplementary Table S3). VespaG consistently outperformed VESPA and ESM-2. Its relative performance w.r.t. the other methods was reasonably stable across all phenotypes (Fig. 2C, Supplementary Table S3). In contrast, SaProt performed much better than the other methods on stability (Δ*ρ* > 0.059), likely due to the fact that it was trained on both sequences and 3D structures, but much worse than all methods except ESM-2 on organismal fitness (Δ*ρ* < −0.066). Overall, predictions for stability were the most accurate across all methods followed by activity, organismal fitness, expression, and finally, binding. We observed a large amplitude between the worst average performance, obtained by ESM-2 on binding (*ρ* = 0.312), and the best one, obtained by SaProt on stability (*ρ* = 0.592).

In addition, assessing VespaG performance in function of mutation depth revealed higher Spearman correlations on single missense variants compared to multiple ones (Supplementary Table S4). We observed a similar trend for all tested predictors, with GEMME consistently yielding the best correlations. Compared to TranceptEVE L, VESPA, and ESM-2, VespaG’s multi-mutant performance was more stable and more closely aligned to its teacher GEMME, especially for mutations of three or more residues.

### 3.2 VespaG integrating complementary strengths

We specifically investigated how VespaG improved over its teacher GEMME and its baseline ESM-2 for exploiting the protein sequence universe, dealing with viral proteins, and handling *de novo* proteins.

Only a small subset of 28 DMS assays from ProteinGym concern viral proteins, including eight from *Influenza A virus*, six from *Human immunodeficiency virus*, four from *bacteriophages*, and two from *SARS-Cov-2* (Supplementary Fig. S3). While VespaG did not match the performance of the top method on this subset (GEMME, Δ*ρ* = −0.048, *P* < 10^−4^), it improved substantially over the ESM-2 baseline (Δ*ρ* = 0.140, *P* < 10^−4^) and the sequence- and structure-based pLM SaProt (Δ*ρ*=0.113, *P* < 10^−3^, Fig. 2B, *PGViral28*; Supplementary Fig. S5). Thus, VespaG was more accurate on viral proteins than other ESM and SaProt versions (Supplementary Figs S8 and S9). This analysis suggests that supervision via GEMME partially counterbalances the poor quality of pLM embeddings for viral proteins. We obtained similar results on a subset of 21 DMS from ProteinGym’s first iteration (Supplementary Fig. S7).

**Figure 3.**
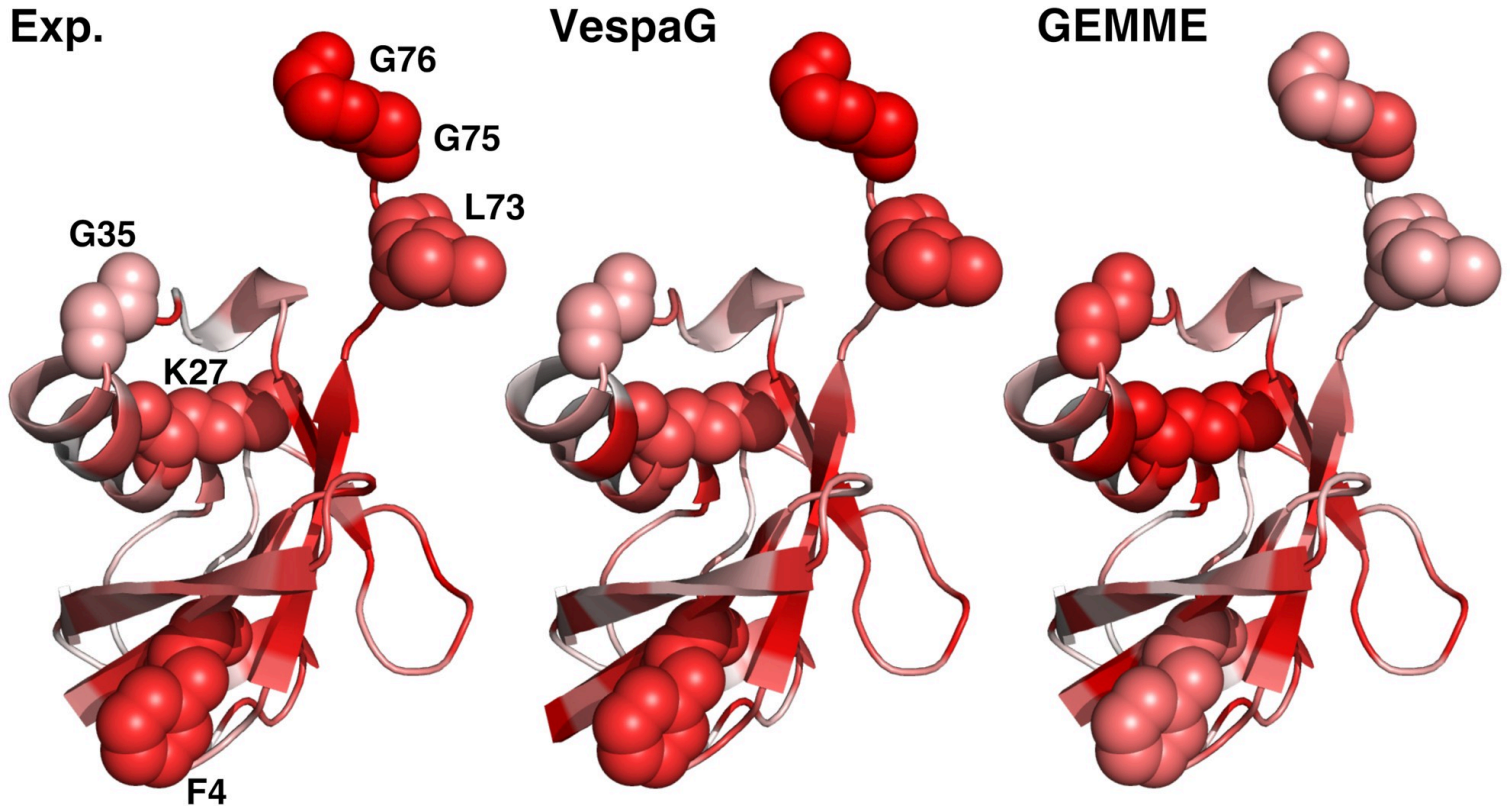
Details of *student* VespaG vs *teacher* GEMME. For the yeast ubiquitin (RL401A_YEAST), we compared experimental measurements (left panel, labeled Exp.; Mavor *et al.* 2016) with predictions by mapping the per-residue mutational sensitivities onto the 3D structures predicted by AlphaFold2 (AF-P0CH08-F1-model_v4, residues 2–76, Jumper *et al.* 2021). We estimated the extent to which a residue is sensitive to mutations as the rank of its average predicted or measured effect over the 19 possible substitutions. The more reddish the more sensitive. We highlighted six residues (labeled by one-letter amino acid code followed by position in the sequence, e.g., G76: Glycine at position 76) for which VespaG agreed with the experiment (rank difference <5) while GEMME strongly disagreed (rank difference >15). The experimental values reflect ubiquitin fitness landscape under normal growth conditions (Mavor *et al.* 2016).

The proverbial student VespaG bested the teacher GEMME by a large margin (Δ*ρ* > 0.1) for the human protein LYAM1, the murine MAFG, the bacterial proteins DN7A, F7YBW7, ISDH, NUSA, and SBI, the plant RCD1, and the yeast ubiquitin RL40A (Supplementary Fig. S5). In particular, VespaG correctly identified the glycines G75 and G76 in the top 5 ubiquitin residues most sensitive to mutations, whereas GEMME incorrectly predicted them as mildly sensitive (Fig. 3). These two residues play essential roles for E1 activation (Mavor *et al.* 2016). Reciprocally, VespaG agreed with the experiment on the mild tolerance of K27, whereas GEMME predicted this residue as highly sensitive (Fig. 3). We can interpret these discrepancies in light of previous works showing that ubiquitin stands out from the general trends between evolutionary sequence conservation and the experimentally measured tolerance to substitutions (Roscoe *et al.* 2013, Mavor *et al.* 2016, 2018). It challenges the common view that high selection pressure implies high mutational sensitivity. Hence, applying this principle on an input MSA, as GEMME does, leads to a limited accuracy (*ρ* in the 0.36–0.44 range). VespaG’s representation learning-based approach allows overcoming this limitation and capturing key aspects of the peculiar sequence-phenotype ubiquitin relationship (*ρ* in the 0.48–0.54 range).

More generally, VespaG has the advantage of being independent of any alignment, whereas GEMME results may substantially differ depending on the chosen MSA generation protocol. Namely, GEMME Spearman correlations displayed large variations (in the [0.1–0.3] range) for 16 assays when retrieving the input MSAs using ColabFold’s MMseqs2-based strategy (Mirdita *et al.* 2022) versus taking the ProteinGym MSAs (Supplementary Fig. S10). The latter were generated with the more sensitive profile hidden Markov model search algorithm JackHMMER (Johnson *et al.* 2010). For almost all these assays (13/16), VespaG achieved a *ρ* value similar to or higher than the maximum *ρ* over the two GEMME runs, regardless of the associated MSA generation protocol (Supplementary Fig. S10). This result suggests that the VespaG framework is at least equivalent to a high-quality MSA-based setup.

Furthermore, VespaG’s independence from alignments makes it applicable to *de novo* proteins. It reached an average Spearman correlation of *ρ*_nov_ = 0.404 ± 0.011 on an additional test set of 146 assays reporting mutation-induced thermodynamic folding stability changes for *de novo* designed 40–72 amino acid long protein domains (Tsuboyama *et al.* 2023) (Supplementary Fig. S11). In contrast, the baseline zero-shot ESM-2, although technically able to handle *de novo* sequences, yielded an extremely poor average Spearman correlation of 0.034. The teacher GEMME only produced predictions for four *de novo* proteins due to a lack of sufficient input alignments. Its Spearman correlation on this small subset was nearly zero (0.085), compared to 0.393 for VespaG.

### 3.3 VespaG generalising across multiple organisms

**Figure 4.**
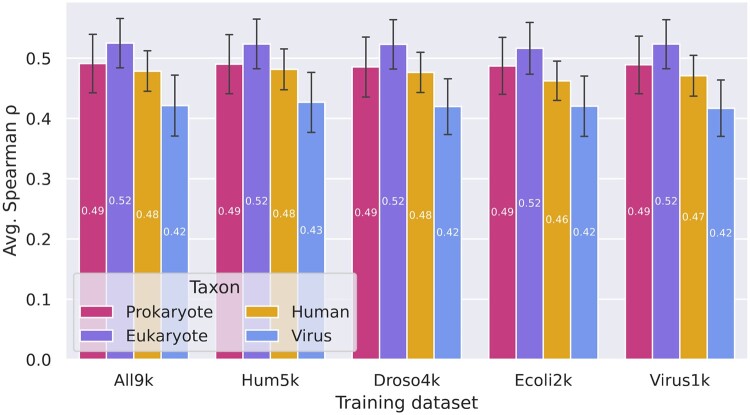
Per-taxon performance of VespaG independent of taxa included in training. For each training set, indicated on the x-axis, we reported the average Spearman correlations computed for each of the five taxa represented in ProteinGym benchmark (Notin *et al.* 2023). The error bars show the 95% confidence interval. Regardless of training data (all data sets were redundancy reduced, names reflect training and bars the test set; *All9k*: about 9000 proteins mixing all taxa shown to the right; *Hum5k*: ∼9000 human proteins, *Droso4k*: ∼4000 fruit fly proteins (*D. melanogaster*), *Ecoli2k*: ∼2000 E.coli proteins (*E. coli*), *Virus1k*: Mix of ∼1500 viral proteins), VespaG generalized equally well for all taxa assessed. For all training sets, it performed best for prokaryotes and non-human eukaryotes, followed by human. Even when trained explicitly on viral proteins, VespaG performed the worst for viral proteins.

We further assessed the impact of the training set on VespaG’s predictive performance and ability to transfer knowledge across organisms. Specifically, we retrained from scratch the same architecture with the same hyperparameters on non-redundant sets of ∼4000 proteins from the insect *D. melanogaster*, ∼2000 proteins from the bacterium *E. coli*, ∼1500 proteins coming from several viruses, and ∼9000 proteins from a combination of all. Training VespaG on a few thousand diverse proteins from a single organism sufficed to generalize across diverse taxa (Fig. 4). Overall, the performance differences for respective taxa were small across organismal training sets (Δ*ρ* ≤ 0.1). However, we consistently observed a lower agreement between VespaG predictions and the experiments for viral proteins, compared to other taxa, across all training sets. In particular, exclusively learning from ∼1500 viral proteins did not improve performance for viral proteins (Fig. 4). Inputting embeddings from other pLMs did not alter this trend (Supplementary Fig. S2).

### 3.4 VespaG predictions blazingly fast

Out of the top performing methods evaluated on the ProteinGym benchmark, VespaG was, in our hands, the most scalable for proteome-wide analyses. Inference on CPU with VespaG needed 5.7 s for the 73 unique proteins from ProteinGym first iteration (Supplementary Fig. S4) on low-end hardware (Intel i7-1355U with 12 × 5 GHz, 1.3 GB RAM, no GPU; Supplementary Table S5). GEMME completed the predictions on the same hardware in 1.27 h (4.2 GB RAM, Supplementary Table S5). Even when considering the time required for input pre-processing, the highly efficient MSA generation of ColabFold could not overcome the runtime advantage of VespaG with a total runtime <1 h on a consumer CPU (Supplementary Table S6). Accessing high-end GPU and CPU resources for pre-processing led to a total execution time of ∼1 min (64.3 s) for VespaG versus ∼90 min (5468.4 s) for GEMME (Supplementary Tables S5 and S6). By comparison, computing zero-shot ESM-2 log-odds took ∼5.35 days and VESPA required 17 h (Supplementary Table S5).

Thus, VespaG was five orders of magnitude faster than ESM-2 (factor 10^5^, i.e. 100 000-times), and three orders of magnitude faster than GEMME and VESPA. The authors of PoET observed their method and TranceptEVE L to be three orders of magnitude slower than GEMME (Truong and Bepler 2023) providing some base to triangulate an estimate between those and VespaG (∼ factor of 10^6^ faster). Additionally, VespaG, unlike other pLM-based methods, does not require a GPU for fast inference (Supplementary Table S5). On a consumer-grade laptop (Supplementary Methods), VespaG computed the entire single-site mutational landscape for a human proteome with 20k proteins (Sinitcyn *et al.* 2023) in fewer than 30 min. On the same machine, at the same time, GEMME completed predictions for 25 proteins. If we assumed that methods such as PoET or TranceptEVE L were executable on low-end hardware, they would have processed 0.025 proteins.

## 4 Discussion

In this work, we explored the possibility of modeling the sequence–phenotype relationship by learning a simple mapping function from protein Language Model (pLM) representations, or embeddings, to evolutionary scores predicted by an expert method. We demonstrated the validity of this approach on several hundred diverse proteins across different organisms. The performance of the resulting method, dubbed VespaG, reached that of much more sophisticated methods.

Using predicted scores instead of curating a dataset of experimental measures allowed the creation of a larger training set (totaling 39M mutations) than those used previously. By comparison, the SNAP2 development set contained about 100 000 mutational effect annotations from 10 000 proteins (Hecht *et al.* 2015). In addition, exploiting only the pLM embedding of the wild-type protein of interest, instead of explicitly modeling its mutants through log-odds probability estimates, enabled reaching a much higher efficiency and inference speed than previous pLM-based methods. The feasibility of this strategy also emphasized the usefulness of the information encoded in a wild-type protein query embedding for assessing all its variants.

### 4.1 VespaG reached SOTA despite its simplicity

The fact that prediction accuracy for VespaG reached the SOTA level proves that even relatively shallow neural networks (660k free parameters) can effectively leverage the knowledge encoded in an unsupervised method such as GEMME (Laine *et al.* 2019). A fundamental difference between student (VespaG) and teacher (GEMME) is its usage of a universal protein representation space. More specifically, VespaG can relate proteins with each other via representations generated by a pLM pre-trained over a huge diversity of natural protein sequences across protein families. This property allows VespaG to generalize across organisms without considering any specific input generation or training schema. Training VespaG on a few thousand proteins from either *H. sapiens*, or *D. melanogaster*, or *E. coli* sufficed to produce high-quality predictions on a diverse set of proteins. For eukaryotic and prokaryotic proteins, the pLM-based student VespaG performed overall numerically higher and more consistently than the MSA-based teacher GEMME. For instance, VespaG improved for cases such as ubiquitin which do not follow the general trends between evolutionary conservation and mutational outcomes. Nevertheless, biases in the pLM representation space may lead to poor predictions for some protein families. Namely, the ProteinGym assessment consistently reported lower accuracy on viral proteins for all zero-shot pLM predictors (Notin *et al.* 2023). Our results demonstrated that supervising on GEMME scores partially counterbalanced this trend. VespaG’s performance decreased for viral proteins, even when explicitly trained on those, but it performed favorably compared to the pLMs ESM-2 and SaProt. Despite retaining high accuracy, GEMME evolutionary scores for viral proteins have a lower resolution than for organismal proteins (Supplementary Fig. S2). Many mutations are assigned the same score, likely reflecting the comparatively lower variability of the associated input MSAs (Supplementary Table S1). Nevertheless, the fact that VespaG trained exclusively on viral proteins exhibits a high predictive capability on organismal proteins (Fig. 4) suggests the impact of this resolution loss is limited. It further supports the hypothesis that the embeddings computed by the pre-trained pLMs for viral proteins are intrinsically noisy. Possibly, viral proteins are simply too under-represented in the training of pLMs, due to a comparatively small number and low diversity (Elnaggar *et al.* 2022, Lin *et al.* 2023, The UniProt Consortium 2023, Ding and Steinhardt 2024). In addition, the pLMs may struggle to capture the inherent peculiarities of viral protein evolution (Koonin *et al.* 2022). Structurally and functionally relevant evolutionary constraints are expected to manifest through smaller differences in viral protein sequences compared to other taxa, warranting a special treatment of these sequences for extracting co-variations (Hopf *et al.* 2017). A future improvement of pLMs could be to develop viral-specific fine-tuning steps. In addition, we showed that VespaG's independence from alignments combined with its inherent generalizability enables tackling *de novo* designed proteins. Most established mutation effect prediction methods, such as GEMME, largely succeed due to evolutionary information derived from MSAs and tend to barely outperform random for single sequences (Hecht *et al.* 2015). Given the absence of reference data for *de novo* proteins, MSA-based tools often fail to provide any result. At the same time, pLMs such as ESM-2 tend to provide unreliable estimates of their respective properties. Although we observed a drop in performance compared to natural proteins, we can envision using VespaG for fast screens before applying future methods adapted to that problem or as a guide for designing more biocompatible *de novo* proteins.

### 4.2 Saving resources as criterion

Although we acknowledge the interest of the pairwise comparison-based predictor ranking scheme introduced recently (Livesey and Marsh 2023), we decided to keep the analysis simpler, tuned to the perspective of the ProteinGym benchmark. Our motivation for this choice is that ranks for individual methods remain short lived although trends for the field appear more stable (Livesey and Marsh 2023, Notin *et al.* 2023). Beyond providing a proof-of-principle for the success of teacher-student strategy in the field of variant effect prediction, our work emphasizes the possibility to improve speed and reduction of energy consumption. VespaG is an extremely fast, cost-efficient, simple tool that invites saving resources at very little cost in terms of accuracy. These properties are highly valuable in a context where many researchers are interested in variant effect predictions for proteomes for which no data is available. Moreover, VespaG’s simplicity stands out in the environment of SOTA predictors. Looking at, e.g. the ablation study of AlphaMissense (Cheng *et al.* 2023), we note how many impressively complex aspects of the method make that method reach its top-level performance. VespaG reaches a similar level without any of that: not using complex machine learning on the side of learning from the teacher, no 3D structure, no MSA, no minor allele frequency-based loss function, no database distillation, asf. Being orders of magnitudes faster than prior methods, VespaG makes it possible, for instance, to explore the effect of a mutation arising in many different contexts such as protein engineering, opening the way to a systematic assessment of epistasis. Additionally, the student−teacher setup of VespaG is easily adaptable to novel pLM input and additional features.

### 4.3 Gain of speed at the expense of interpretability?

GEMME reaches its SOTA-level performance by optimizing only two simple parameters: The conservation of a position in a family of related proteins and the distance of a variant on the tree. Simply put, GEMME predicts effect when variants deviate from the observed conservation pattern and neutral when the variants have been observed close on the tree. *Per se*, VespaG has no such interpretability. As it learned from GEMME, users can replace “strongly predicted effect” to imply variant against conservation even without seeing the MSA, and conversely “strongly predicted neutral” as examples observed nearby on the tree. In fact, in contrast to GEMME, VespaG quantifies the strength of the prediction. This in itself seems an important feature relevant for users. For the analysis of particular variants, users might want to actually generate MSAs and trees on their own to support their rationales. However, neither any of the two, nor—to the best of our knowledge—any of the other SOTA methods, directly generate a hypothesis for how a variant may disrupt the details of molecular function.

### 4.4 Interpretable models of variant effects?

For some particular applications, users might be interested to have the “molecular” or “mechanical” explanation why a particular variant affects protein function or why it is neutral. Future efforts may aim at more explicitly encoding evolutionary semantics in the pLM representation space, e.g., by exploiting GEMME intermediate results (evolutionary distances). Modeling and tracing the evolutionary history of natural sequences in that space could provide the key to pinpoint gain-of-function mutations and distinguish them from loss-of-function ones, a major current challenge for the field. Another direction for future improvements could be the combination of effect prediction methods, such as GEMME, or VespaG, with methods optimized to predict certain phenotypes as has been proposed before (Ioannidis *et al*. 2016). For instance, many methods predicting thermodynamic stability changes (Benevenuta *et al*. 2023) exploit complementary information about 3D structures, and the expert-guided ML method RaSP already enables large-scale screens (Blaabjerg *et al*. 2023). Ideally, the field would advance by adding semi-automated tools to turn predictions into mechanical explanations using annotations and predictions of protein structure and function.

In conclusion, VespaG closes the gap in performance between the best and the fastest missense amino acid variant effect predictors. For an unprecedentedly small trade-off in performance, it can predict variants several orders of magnitude faster than other state-of-the-art methods.

## Supplementary Material

btae621_Supplementary_Data
