# Supplementary material for: Expert-guided protein language models enable accurate and blazingly fast fitness prediction
Source: Bioinformatics. 2024 Nov 22;40(11):btae621. doi: 10.1093/bioinformatics/btae621 (PMC11588025; doi:10.1093/bioinformatics/btae621)
Supplement: btae621_Supplementary_Data [file btae621_supplementary_data.pdf]

Supporting Online Material

Expert-guided protein Language Models enable accurate and blazingly fast fitness prediction

Céline Marquet[1,✧,*], Julius Schlensok[1,✧], Marina Abakarova[2,5], Burkhard Rost[1,3,4] & Elodie Laine [2,6,*]

1 TUM (Technical University of Munich), Germany; TUM School of Computation, Information and Technology; Department of Informatics, Bioinformatics & Computational Biology - i12, Boltzmannstr. 3, 85748 Garching/Munich, Germany

2 Sorbonne Université, CNRS, IBPS, Laboratory of Computational and Quantitative Biology (LCQB), UMR 7238, 75005 Paris, France

3 Institute for Advanced Study (TUM-IAS), Lichtenbergstr. 2a, 85748 Garching/Munich, Germany

4 TUM School of Life Sciences Weihenstephan (TUM-WZW), Alte Akademie 8, Freising, Germany

5 Universite Paris Cité, INSERM UMR U1284, 75004 Paris, France

6 Institut universitaire de France (IUF)

* Corresponding authors: celine.marquet@tum.de, elodie.laine@sorbonne-universite.fr

✧ Céline Marquet and Julius Schlensok contributed equally to this work

Description

This supporting material provides information to help the reader with reproducing and re-interpreting the results of the main article on VespaG, a blazingly fast amino acid variant effect predictor, leveraging embeddings of protein language models (pLMs) as input to a minimal deep learning model. Supplementary Methods give an overview of related works and some details about the evaluation procedure. Tables S1-S2 and Figures S1-S2 highlight method development w.r.t. hyperparameter configurations and pLM types. Figures S3 and S4 show test set properties. Tables S2-S3 and Figures S5-S11 present more details on the Spearman correlation between experimental results and evaluated methods. Tables S4-S5 highlight runtime performance of evaluated methods.

TABLE OF CONTENTS

1. Supplementary Methods.
2. Table S1: Size statistics of training datasets used.
3. Table S2: Hyperparameters and number of free parameters.
4. Table S3: Spearman Correlation ρ between ProteinGym substitution benchmark and SOTA methods.
5. Table S4: Spearman Correlation ρ with ProteinGym substitution benchmark for methods grouped by number of point mutations in mutants.
6. Table S5: Runtime comparison for VespaG, GEMME, VESPA, and ESM-2 (3B) inference.
7. Table S6: Runtime comparison for VespaG, GEMME, and VESPA pre-processing steps.
8. Figure S1: Influence of the architecture and the input embeddings on VespaG's validation loss.
9. Figure S2: Influence of the training dataset and the input embeddings on VespaG's validation loss.
10. Figure S3: Properties of the ProteinGym substitution benchmark set.
11. Figure S4: Properties of the first iteration of the ProteinGym substitution benchmark set.
12. Figure S5: Average Spearman correlation of VespaG and SOTA methods on unique proteins of *ProteinGym217.*
13. Figure S6: Consensuality of the predictions on ProteinGymOrganismal189.
14. Figure S7: Average Spearman correlation of VespaG and SOTA methods on the first iteration of ProteinGym.
15. Figure S8: Average Spearman correlation of VespaG and SOTA methods on 189 organismal DMS assays of the ProteinGym benchmark.
16. Figure S9: Average Spearman correlation of VespaG and SOTA methods on 28 viral DMS assays of the ProteinGym benchmark.
17. Figure S10: Comparison of Spearman correlation depending on the input alignment.
18. Figure S11: Average Spearman correlation between predicted mutational effect scores and experimental ΔΔG scores.
19. References

Supplementary Methods

Related work

A key component of computational variant effect predictors is the ability to capture arbitrary range dependencies between amino acid residues. A majority of predictors extracts these dependencies from an input multiple sequence alignment (MSA) generated from large protein sequence databases. Some rely on the statistical inference of pairwise couplings (Figliuzzi et al. 2016; Hopf et al. 2017), others on the implicit account for global context with latent variables (Frazer et al. 2021; Riesselman, Ingraham, and Marks 2018). They remain computationally costly due to the large number of inferred parameters and strongly depend on the input alignment's variability.

More recently, using representations generated by protein language models (pLMs) emerged as an alternative to using MSAs as input (Brandes et al. 2023; Cheng et al. 2023; Marquet et al. 2022; Meier et al. 2021; Nijkamp et al. 2022; Notin, Dias, et al. 2022). These high-capacity pLM transformer architectures, borrowed from natural language processing, learn to reconstruct masked or missing amino acids in an input query sequence (Elnaggar et al. 2021; Lin et al. 2023). They model raw protein sequence data over large databases, thereby capturing evolutionary constraints that generalize across protein families. Once trained, they can serve as zero-shot variant effect predictors by estimating the likelihood of each amino acid at each position.

A limitation of pLMs is that they overlook natural protein sequences' evolutionary history and relationships. Strategies for overcoming this oversimplification aim at encoding evolutionary semantics in the pLMs representation space, *e.g.*, by augmenting the input with a multiple sequence alignment (Rao et al. 2021). The latter informs the model about sequences evolutionary related to the input query and how their amino acids match. The integration of weak labels coming from inter-individual polymorphisms and of a physical prior through supervised learning of the 3D structure, as AlphaMissense does, further enhances the predictive performances (Cheng et al. 2023). Alternatively, the predictor VESPA combines the pLM-derived log-odds substitution scores with evolutionary conservation levels predicted from the learned protein representations (Marquet et al. 2022). The predictors Tranception (Notin, Dias, et al. 2022) and PoET (Truong Jr and Bepler 2023) have also explored retrieval-augmented strategies by conditioning the predictions of a pre-trained pLM on a set of related raw or aligned protein sequences at inference time. Others successfully included structural information to enhance performance for various supervised and unsupervised downstream prediction tasks (Heinzinger et al. 2024; Su et al. 2024; Tan et al. 2024).

Comparison to State-of-the-art methods

We computed VESPA and GEMME predictions for both test sets, while we downloaded TranceptEVE L, SaProt and ESM-2 predictions from ProteinGym. For PoET, the authors provided Spearman correlation values for each experimental assay in the first iteration of the ProteinGym test set on request. For AlphaMissense, we extracted pre-computed per-assay scores of the first ProteinGym iteration from the supplemental materials. Due to the different ways of accessing or computing the predictions of GEMME, TranceptEVE, PoET, and AlphaMissense, we did not evaluate the effect of using different MSAs as input. GEMME predictions were generated through the MMseqs2-based ColabFold protocol as described in (Abakarova et al. 2023). Further, we did not benchmark ensemble methods such as PoET+GEMME (Truong Jr and Bepler 2023) on the test sets as ensembling any other method with VespaG would eradicate its speed advantage. To assess thermodynamic folding stability of de novo domains, we only compared VespaG with ESM-2 and GEMME, since precomputed results were not available for the other methods.

Method development

Datasets

Test data

Protein sequences and experimental scores were accessed from the ProteinGym GitHub repository (Notin 2024). The ProteinGym substitution benchmark was not redundancy reduced, and we divided the ProteinGym into subsets based on organisms, function, and time of availability. The first split by organism can contain any function: (1) *PGOrganismal189* with 189 assays on 161 prokaryotic and eukaryotic proteins, and (2) *PGViral28* with 28 assays on 26 viral proteins. The second split was based on function and can contain prokaryotic, eukaryotic and viral proteins: (3) *PGStability66* with 66 assays assessing stability, (4) *PGActivity43* with 43 assays assessing activity, (5) *PGBinding13* with 13 assays assessing binding, (6) *PGExpression18* with 18 assays assessing organismal expression, and (7) *PGFitness77* with 77 assays assessing fitness. The last splits were added to assess methods for which predictions were available only for the first iteration of ProteinGym. The first iteration was divided into (8) *PGOrganismal66,* containing 66 assays of 54 proteins with 1.4M variants, and (9) *PGViral21*, containing 21 assays of 19 proteins with 184K variants.

Training data

To redundancy reduce the training data, we clustered the protein sequences using UniqueProt (Mika 2003) with an HSSP (homology-derived secondary structure of proteins)-value < 0, corresponding to no pair of proteins in the redundancy reduced data

set having over 20% pairwise sequence identity over 250 aligned residues (Rost 1999; Sander and Schneider 1991). This criterion is stricter than the classically used threshold of 30% overall sequence identity. It accounts for the fact that protein structures can show high similarity even at lower sequence similarity levels (Rost 1999). To improve runtime, we modified the original UniqueProt protocol (Olenyi, Tobias et al. n.d.) by replacing BLAST (Altschul et al. 1997) with MMseqs2 (Steinegger and Söding 2017). Additionally, we discarded alignments of fewer than 50 residues for pairs of sequences with more than 180 residues as they provide only a relatively weak support for similarity.

Model Specifications

Configuration

All layers were linked through the LeakyReLu activation function (Agarap 2019; Xu et al. 2015), as well as dropout (Srivastava et al. 2014). No activation function was used for the output layer. We implemented the models in PyTorch (Paszke et al. 2019) v1.13.1 using Python 3.10.8. They were trained using the AdamW (Kingma and Ba 2017; Loshchilov and Hutter 2019) optimizer with an initial learning rate of 10e-4, decaying with a decay factor of 0.33 and a patience of 8 epochs using mean squared error (MSE) loss between predicted and target GEMME scores. Each batch contained 25K residues. We applied early stopping based on the validation partition loss, with a patience of 10 epochs. The maximal training duration was 200 epochs. Hyperparameters were optimized through exhaustive parameter search on the validation split of the *Hum5k* dataset for each architecture. The same hyperparameters were then used to re-train separate models on each of the additional datasets (Droso4k, Ecoli2k, Virus1k, and All9k), using the same training scheme (SOM Table S1). The training dataset and hyperparameters of the final model were selected based on validation loss.

Multi-mutations

To score combinations of multiple substitutions, we add the scores of their constituent single substitutions, a technique introduced previously (Meier et al. 2021). For instance, in the case of the double mutation *M1A:G2N*, i.e. the mutation of a Methionine to an Alanine at the first residue of the protein and a Glycine to an Asparagine at the second, the score of the double mutation is the sum of the computed scores of the single mutations *M1A* and *G2N*.

Score Transformation

To ease score interpretability, VespaG scores are transformed to the same distribution as GEMME scores by linearly interpolating raw VespaG scores based on their quantile compared to a distribution of raw VespaG scores from a set of 10k mutations for which GEMME scores are known, resulting in scores ranging in the [-10, 2] interval. To further

limit them to [0, 1], a sigmoid transformation is applied to the transformed scores. Both transformations can be individually toggled off by the user if desired.

Evaluation

Performance measures

To assess the performance of the predictors, we relied on the Spearman rank correlation coefficient: $\rho_s = r_{R(X),R(Y)} \frac{cov(R(X),R(Y))}{\sigma_{R(X)}\sigma_{R(Y)}}$, with raw scores X, Y as ranks $R(X), R(Y)$, Pearson correlation coefficient $r_{R(X),R(Y)}$, covariance $cov(R(X), R(Y))$, and standard deviations $\sigma_{R(X)}$, $\sigma_{R(Y)}$. This metric enables quantifying the strength of non-linear relationships between predicted and experimental scores. The Spearman correlation coefficient was computed for each DMS experiment separately. For proteins covered by multiple DMS experiments, we averaged the correlation over the experiments before computing the mean over all proteins.

Error estimates

To assess the significance of the performance differences between the evaluated predictors, we computed symmetric 95% confidence intervals (CI) $\mu \pm 1.96 * SEM$, with $SEM = \frac{\sigma}{\sqrt{n}}$ as the Standard Error of the Mean over n = 1,000 bootstraps using sampling with replacement from the respective datasets. The terms μ and σ are the mean and standard deviation of the bootstrap distribution, respectively. In addition, we performed one-tailed paired t-tests on the distributions of Spearman rank correlation coefficients. For any pair of methods, the test statistic is expressed as, $t = \frac{\bar{d}}{\sigma/\sqrt{n}}$, where $\bar{d}$ and σ are the mean and standard deviation of the n correlation coefficient differences. The associated p-value reflects the probability of observing the test statistic under the null hypothesis (no significant difference, the true mean of differences is zero). It is obtained by comparing t to a t-distribution with n-1 degrees of freedom.

Baseline

To compare model performances on the validation set to an untrained random baseline retaining on the target distribution, we randomly permuted the GEMME target scores for each protein, i.e. randomly shuffled all values of the Lx20 GEMME output matrix.

Runtime

We measured the wall-clock runtime of VESPA, GEMME, and VespaG for 1.6M mutations of the 73 unique proteins of the first iteration of the ProteinGym test set using 32 CPU cores of an Intel Xeon Gold 6248 at 2.50 GHz and 64 GB of DDR4 ECC RAM. VESPA, in addition, was allotted an Nvidia Quadro RTX 8000 GPU with 48 GB VRAM. Runtimes for other methods could not be obtained due to computational limitations. We did not measure the runtime for the generation of the input MSAs for GEMME, nor the input embeddings for the embedding-based methods since we used pre-computed data.

Name	Source organism(s)	Number of sequences				Number of residues	
		Reference proteome	After Length restriction (Min.25, Max.1024)	After Redundancy Reduction	Confident GEMME predictions	Total	Min., Max., Median protein length
**Hum5k**	*Homo sapiens*	20,357	18,043	5,886	5,305	2,010,031	36 / 1024 / 328
**Droso4k**	*Drosophila melanogaster*	13,811	12,305	4,809	4,081	1,610,079	40 / 1024 / 346
**Ecoli2k**	*Escherichia coli*	4,403	4,284	2,544	2,333	652,257	36 / 990 / 243
**Virus1k**	All viral in Swiss-Prot	17,320	15,954	4,498	1,400	401,204	29 / 1017 / 228
**All9k**	All of above	55,891	50,586	15,175	9,616	3,291,539	29 / 1024 / 286

Table S1: **Size statistics of training datasets used.** The dataset used for the development of VespaG, dubbed Hum5k, is a redundancy reduced version of the human proteome. To investigate generalizability across organisms, we further analyzed the performance of VespaG trained on several other datasets. These included the redundancy reduced proteomes of *Drosophila melanogaster* (Droso4k) and *Escherichia coli* (Ecoli2k), a redundancy reduced set of all viral proteins in Swiss-Prot (Virus1k), and a redundancy reduced combination of all (All9k).

Model	Best hyperparameters	# free parameters (ProtT5/ESM-2)*
LinReg	Dropout of 0.4	1025 / 2561
**FNN_1_layer (*VespaG*)**	Hidden layer with size 256, dropout 0.2.	267k / 660k
FNN_2_layer	Hidden layers of sizes 256 and 64 without dropout.	280k / 673k
CNN	1D convolution from input to 256 channels with kernel size 7 and padding 3 with dropout rate 0.2. Fully-connected hidden layers of size 256 and 64 without dropout.	1.91m / 4.67m
FNN+CNN mean	FNN_2_layer + CNN	2.19m / 5.34m

Table S2: **Hyperparameters and number of free parameters.** We built the predictors with 5 architectures, for each the selected hyperparameters and number of free parameters is listed. Hyperparameters were optimized through exhaustive parameter search on the validation split of the *Hum5k* dataset for each architecture. Methods are: (1) Linear regression, *i.e.*, a feed-forward neural network (FNN) without any hidden layer, dubbed *LinReg*; (2) FNN with one hidden layer, called *FNN_1_layer (VespaG)*; (3) FNN with two hidden layers, called *FNN_2_layer*; (4) Convolutional neural network (CNN) with one 1-dimensional convolution and two hidden dense layers, referred to as *CNN*; and (5) an ensemble of separately optimized FNN and CNN (with the same architecture as the best stand-alone model for each architecture), with the output being the mean of the two networks.
*Size of input embeddings: ProtT5 1024xL and ESM-2 2560xL

ProteinGym Subset	VespaG	GEMME	TranceptEVE L	VESPA	ESM-2 3B	SaProt 650M
PG217 (averaged per-protein)	0.480 ±0.021	**0.486 ±0.021**	0.474 ±0.021	0.460 ±0.02 0	0.430 ±0.028	0.472 ±0.027
PG217 (weighted average per-function)	0.459 ±0.050	**0.464 ±0.041**	0.456 ±0.040	0.436 ±0.043	0.406 ±0.057	0.457 ±0.074
PGOrganismal189	0.491 ±0.024	0.490 ±0.024	0.478 ±0.023	0.464 ±0.023	0.455 ±0.027	**0.500 ±0.026**
PGViral28	0.414 ±0.049	**0.462 ±0.045**	0.453 ±0.052	0.432 ±0.045	0.274 ±0.087	0.300 ±0.083
PGActivity43	**0.494 ±0.043**	0.493 ±0.045	0.487 ±0.045	0.468 ±0.043	0.417 ±0.061	0.458 ±0.055
PGBinding13	0.370 ±0.086	**0.397 ±0.090**	0.376 ±0.085	0.366 ±0.076	0.321 ±0.103	0.379 ±0.075
PGExpression18	0.456 ±0.063	0.443 ±0.066	0.457 ±0.065	0.404 ±0.067	0.403 ±0.081	**0.488 ±0.064**
PGFitness77	0.441 ±0.036	**0.460 ±0.034**	**0.460 ±0.034**	0.440 ±0.035	0.379 ±0.048	0.367 ±0.046
PGStability66	0.533 ±0.036	0.528 ±0.038	0.500 ±0.040	0.500 ±0.037	0.509 ±0.040	**0.592 ±0.033**

Table S3: Spearman correlation coefficient ρ between predicted and experimental substitution effect scores on ProteinGym substitution benchmark (with several subsets) for methods VespaG, GEMME (Laine et al., 2019), TranceptEVE L (Notin et al., 2022), VESPA (Marquet et al., 2022), ESM-2 (3B) (Lin et al., 2023), and SaProt (650M) (Su et al., 2024). Cells show mean ρ ± standard error for subsets: *ProteinGym217 (per-protein)* - all 217 DMS, weighted by the number of DMS per protein, *ProteinGym217 (per-function)* - all 217 DMS, weighted by the number of DMS per protein and the number of assays per category. All others are weighted by the number of DMS per protein: *ProteinGymOrganismal189* - 189 eukaryotic and prokaryotic DMS,

ProteinGymViral28 - 28 viral DMS, *ProteinGymActivity43* - 43 DMS on activity, *ProteinGymBinding13* - 13 DMS on binding, *ProteinGymExpression18* - 18 DMS on expression, *ProteinGymFitness77* - 77 DMS on organismal fitness, and *ProteinGymStability66* - 66 DMS on stability. Numerically highest values per row highlighted in bold. Standard error bootstrapped over unique proteins for all subsets except *ProteinGym217 (per-function)* where it was instead bootstrapped over the 5 functional categories.

Mutational Depth	Distribution		Avg. Spearman ρ				
	# assays	# mutations	VespaG	GEMME	Trancept EVE L	VESPA	ESM-2 (3B)
1	217	696,311	0.462	**0.464**	0.391	0.396	0.366
2	69	826,245	0.249	**0.292**	0.256	0.183	0.208
3	11	84,134	0.347	**0.376**	0.250	0.324	0.179
4	11	187,850	0.319	**0.349**	0.211	0.278	0.150
5+	9	671,227	0.367	**0.423**	0.249	0.287	0.185

Table S4: **Spearman correlation coefficient ρ between predicted and experimental substitution effect scores on ProteinGym substitution benchmark partitioned by mutational depth.** We report results for methods VespaG, GEMME (Laine et al., 2019), TranceptEVE L (Notin et al., 2022), VESPA (Marquet et al., 2022), and ESM-2 (3B) (Lin et al., 2023). Numerically highest values per row highlighted in bold. Results were not averaged by protein or assay type. No per-mutant predictions for SaProt were available to download via ProteinGym as of June 2024.

Inference			
**Tool**	**Hardware**	**Runtime [s]**	**Memory usage**
VespaG	Consumer CPU	5.7	1.3 GB
GEMME	Consumer CPU	4,561.4	4.2 GB
ESM-2 (3B)<br>log-odds ratios	High-end GPU	462,417.34	48 GB
VESPA	High-end GPU	63,693.0	48 GB

Table S5: Runtime of VespaG, GEMME, ESM-2 (3B) and VESPA inference on 73 unique proteins of the first iteration of the ProteinGym substitution benchmark (Notin et al., 2023) with precomputed input. Hardware used: consumer CPU - Intel i7-1355U with 12x5 GHz, high-end GPU - Nvidia Quattro RTX 8000 or Nvidia RTX 6000 (both 48GB VRAM). Methods benchmarked: VespaG with pre-computed ESM-2 (3B) per-residue embeddings, GEMME with pre-computed ColabFold alignments (Laine et al., 2019; Mirdita et al., 2022), VESPA (Marquet et al., 2022) with pre-computed ProtT5 per-residue embeddings, and ESM-2 (3B) log-odds scores (Lin et al., 2023). For the runtimes of pre-processing steps, see Tab. S4b.

Preprocessing				
**Tool**	**Step**	**Hardware**	**Runtime [s]**	**Memory usage**
VespaG	Embedding generation (ESM2)	High-end GPU	58.6	48 GB
		Consumer CPU	3,226.7	20.1 GB
		High-end CPU	200.73	24 GB
VESPA	Embedding generation (ProtT5)	High-end GPU	53.1	48 GB
GEMME	ColabFold MSA generation	High-end CPU	870.0	17.5 GB
	MSA preprocessing	High-end CPU	37.0	17.5 GB
	Total	High-end CPU	907.0	17.5 GB

Table S6: Runtime of preprocessing steps for methods VespaG, VESPA, and GEMME on 73 unique proteins of the first iteration of the ProteinGym substitution benchmark (Notin et al., 2023). Hardware used: Consumer CPU: Intel i7-1355U with 12x5 GHz, high-end CPU: 4 cores of a shared AMD EPYC Milan with 32x2.95 GHz, high-end GPU: Nvidia Quattro RTX 8000 or Nvidia RTX 6000 (both 48GB VRAM). The three values reported for VespaG embedding generation correspond to three independent runs on three different kinds of hardware.

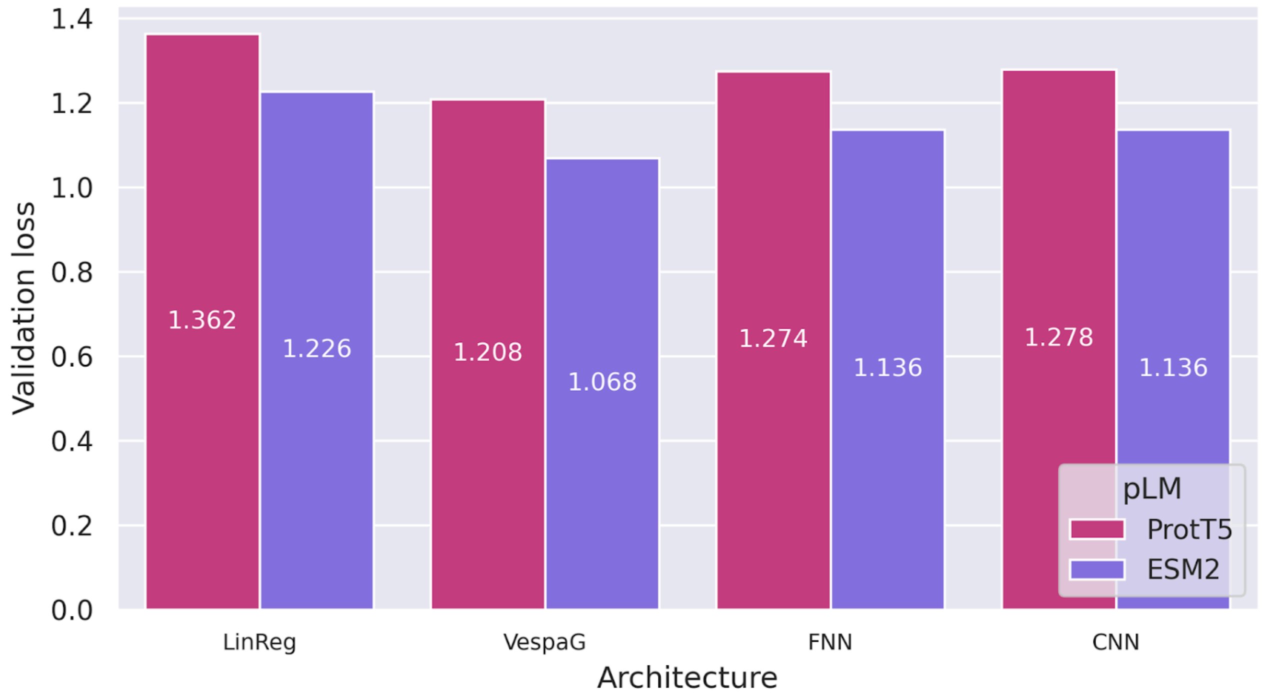

Figure S1: Influence of the architecture and the input embeddings on VespaG's validation loss. The loss (mean squared error (MSE) between predicted and target GEMME (Laine et al., 2019) scores) is computed on the randomly selected validation split of the *Hum5k* dataset. ESM-2 embeddings (Lin et al., 2023) yield higher performance than ProtT5 (Elnaggar et al., 2021) across all architectures.

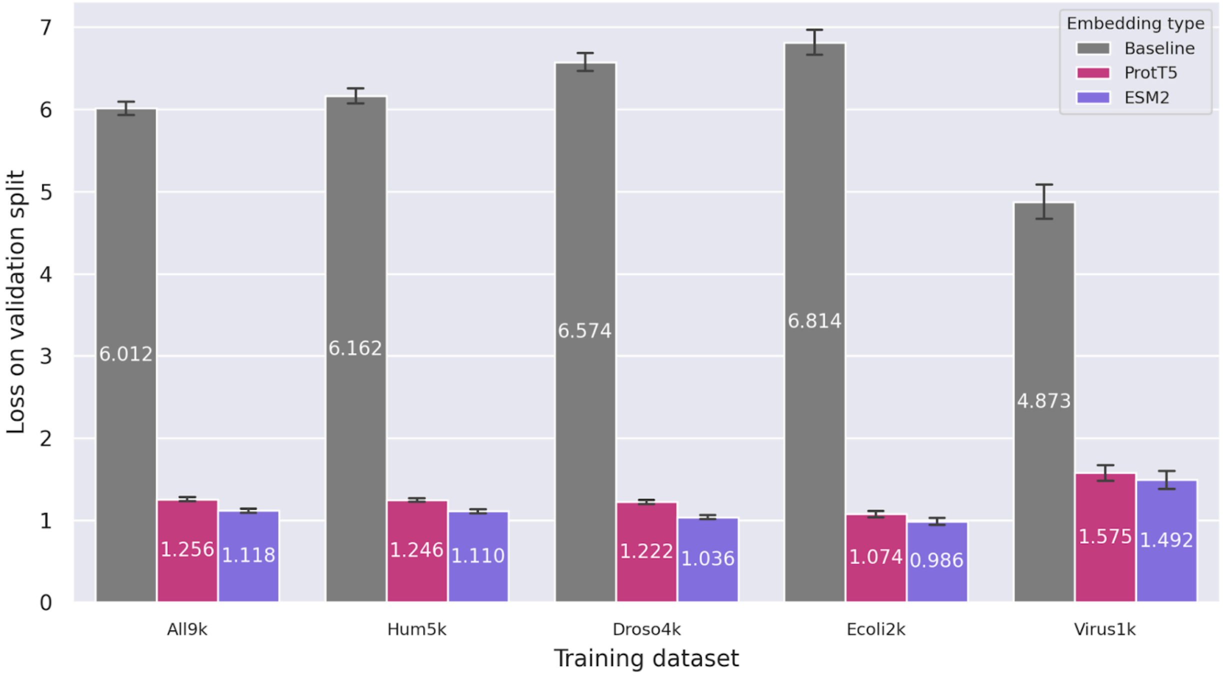

Figure S2: Influence of the training dataset and the input embeddings on VespaG's validation loss. The loss (mean squared error (MSE) between predicted and target GEMME (Laine et al., 2019) scores) is computed on the randomly selected validation split of each training dataset and compared to a baseline obtained with randomly permuted GEMME scores (see Materials and Methods). ESM-2 embeddings (Lin et al., 2023) yield significantly higher performance than ProtT5 (Elnaggar et al., 2021) in all cases except the viral training dataset. Both types of embeddings lead to degraded performance on this dataset, whereas the performance of the random baseline is comparatively much better. This observation suggests that GEMME scores have a much lower resolution on viral proteins (more identical or very similar scores). Error bars show 95% confidence intervals.

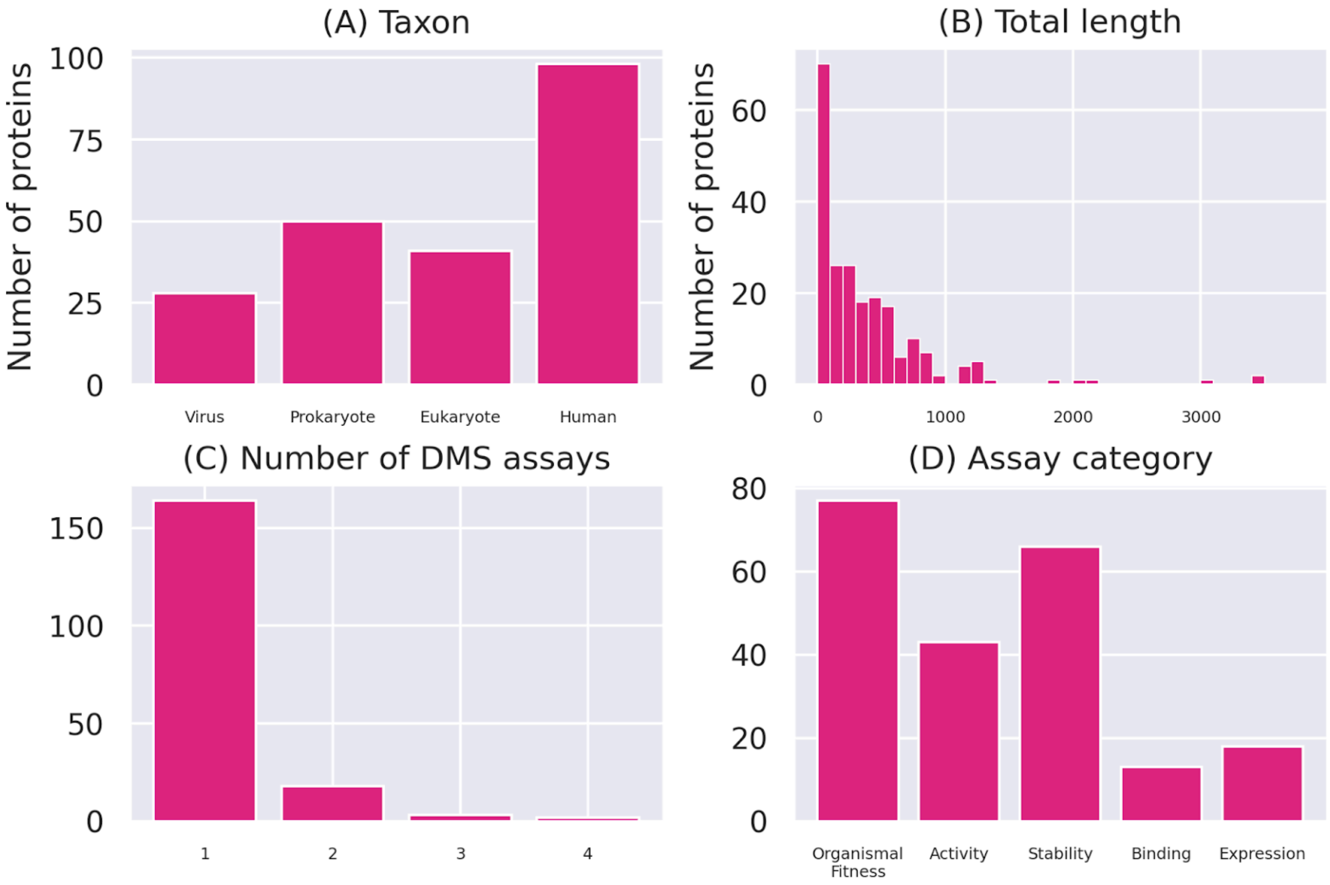

Figure S3: **Properties of the ProteinGym substitution benchmark set** (Notin et al., 2023). (A) Distribution of proteins across taxa (Eukaryote referring to non-Human eukaryotic proteins) (B) Distribution of sequence length per protein (C) Distribution of number of deep mutational scanning (DMS) assays per protein (D) Distribution of assay categories.

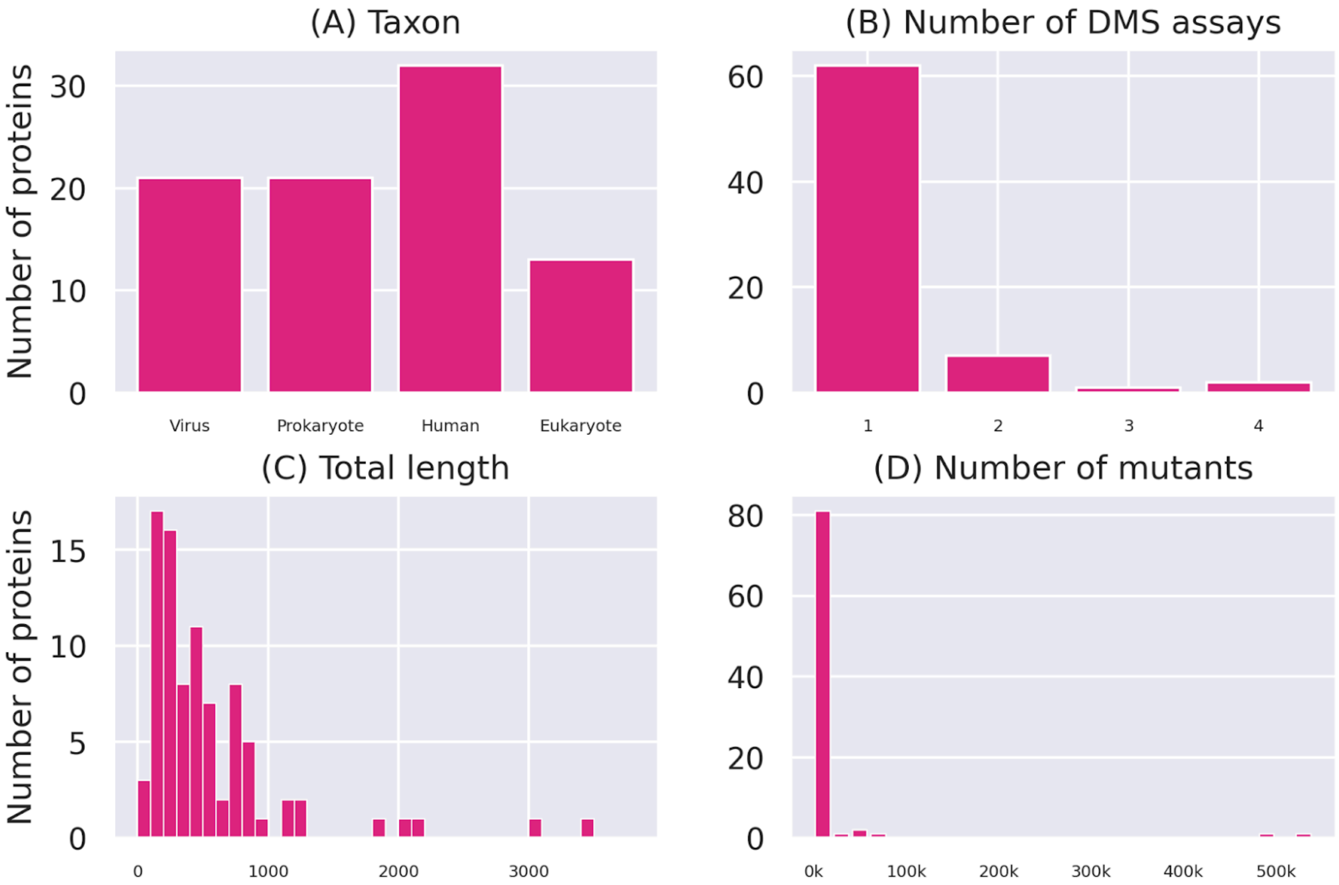

Figure S4: **Properties of the first iteration of the ProteinGym substitution benchmark set** (Notin et al., 2023). (A) Distribution of proteins across taxa (Eukaryote referring to non-Human eukaryotic proteins) (B) Distribution of number of deep mutational scanning (DMS) assays per protein (C) Distribution of sequence length per protein (D) Distribution of number of mutants across DMS assays.

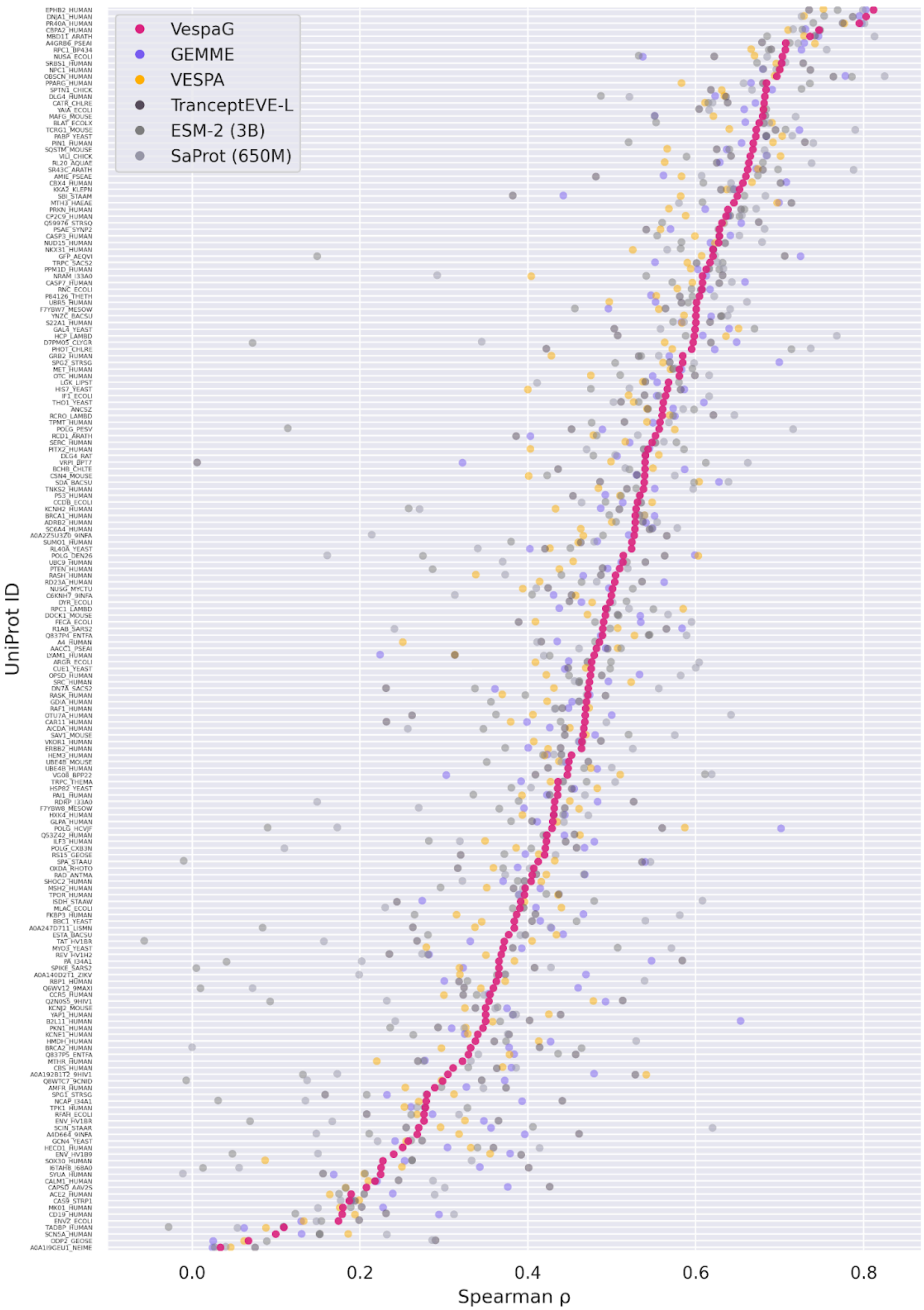

Figure S5: Average Spearman correlation coefficient ρ between predicted and

experimental substitution effect scores for VespaG and SOTA methods on unique proteins of *ProteinGym217* ordered by VespaG performance. Other methods reported are GEMME (Laine et al., 2019), TranceptEVE L (Notin et al., 2022), VESPA (Marquet et al., 2022), ESM-2 (3B) (Lin et al., 2023), and SaProt (650M) (Su et al., 2024). Spearman correlations of methods depicted in shades of gray (TranceptEVE L, ESM-2 and SaProt) were downloaded from the ProteinGym website.

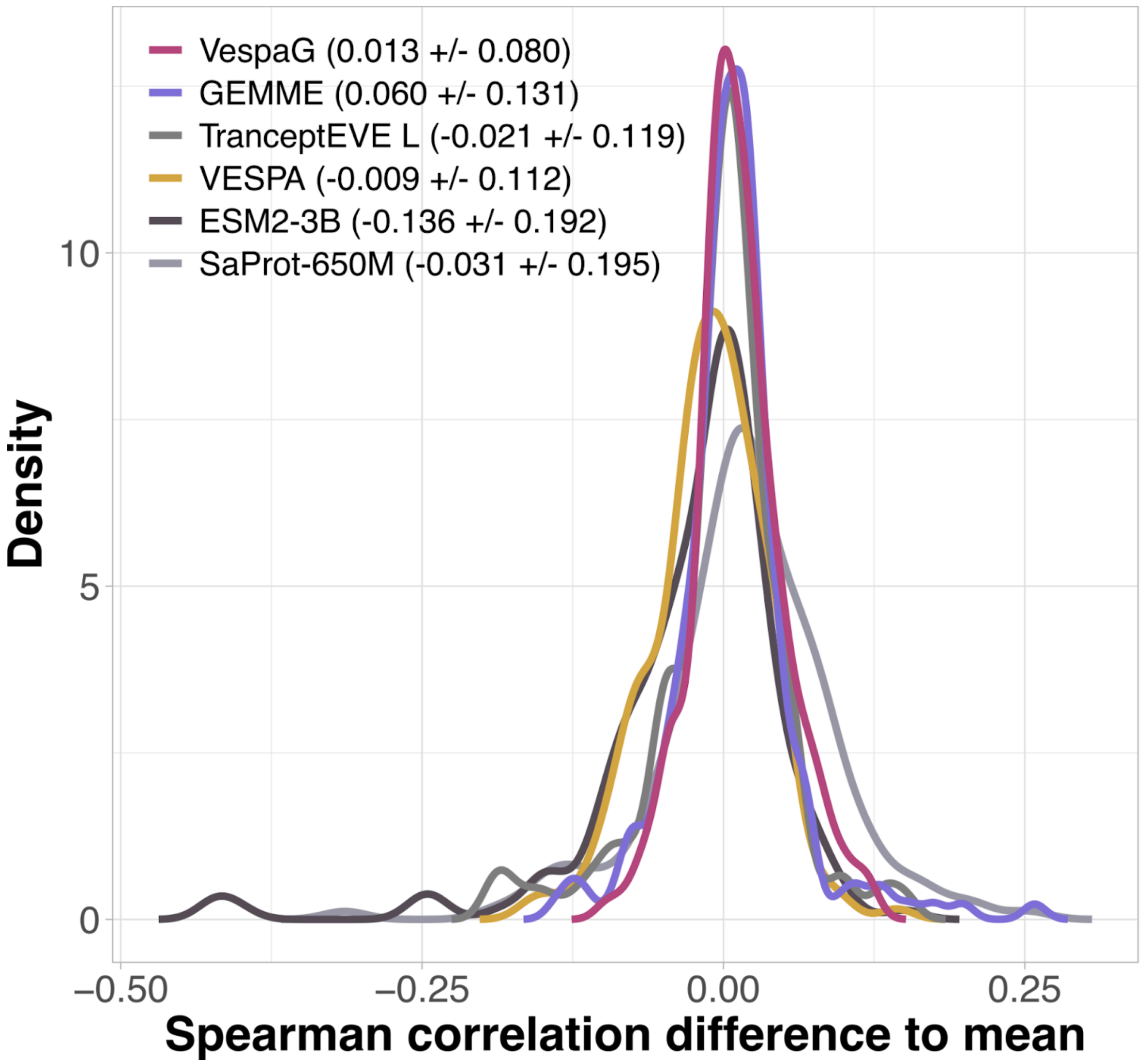

Figure S6: Consensuality of the predictions on ProteinGymOrganismal189. For each predictor, we report the distribution of the differences between its performance values (Spearman correlation coefficients with experiments) and the average performance of all six highlighted predictors on the *ProteinGymOrganismal189* test set. The mean and standard deviation for each density are indicated in parenthesis.

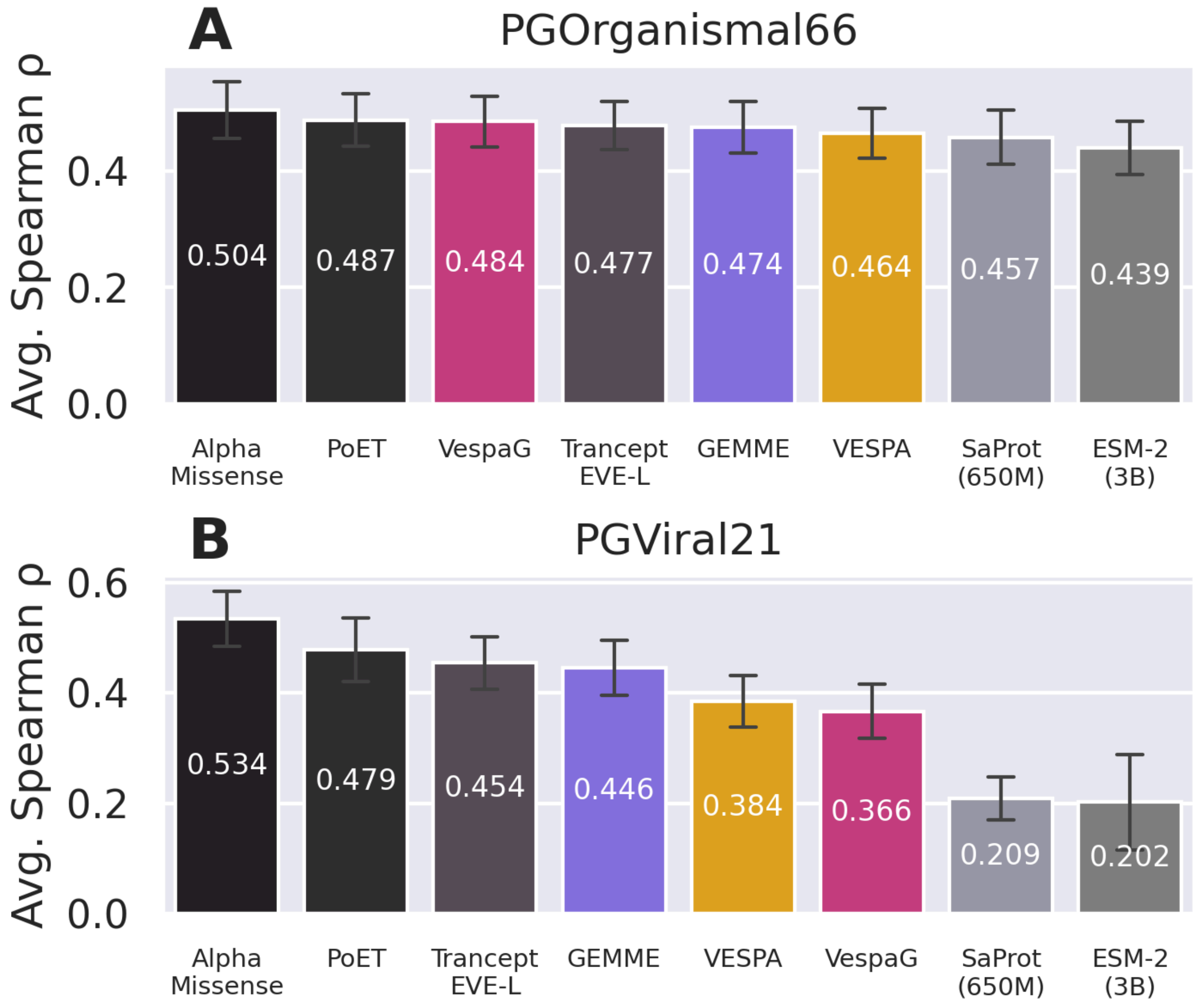

Figure S7: Average Spearman correlation coefficient ρ between predicted and experimental substitution effect scores of VespaG and SOTA methods on the first iteration of ProteinGym. Methods presented are VespaG, GEMME (Laine et al., 2019), TranceptEVE L (Notin et al., 2022), VESPA (Marquet et al., 2022), ESM-2 (3B) (Lin et al., 2023), SaProt (650M) (Su et al., 2024), AlphaMissense (Cheng et al., 2023), and PoET (Truong Jr and Bepler, 2023). Results for methods depicted in gray were downloaded from ProteinGym (TranceptEVE L, ESM-2 and SaProt) or taken from the respective publications (AlphaMissense, PoET). Panel (A) *ProteinGymOrganismal66*, containing 66 experimental assays for 54 eukaryotic and prokaryotic proteins; (B) *ProteinGymViral21*, containing 21 assays for 19 viral proteins of the first iteration of the ProteinGym substitution benchmark.

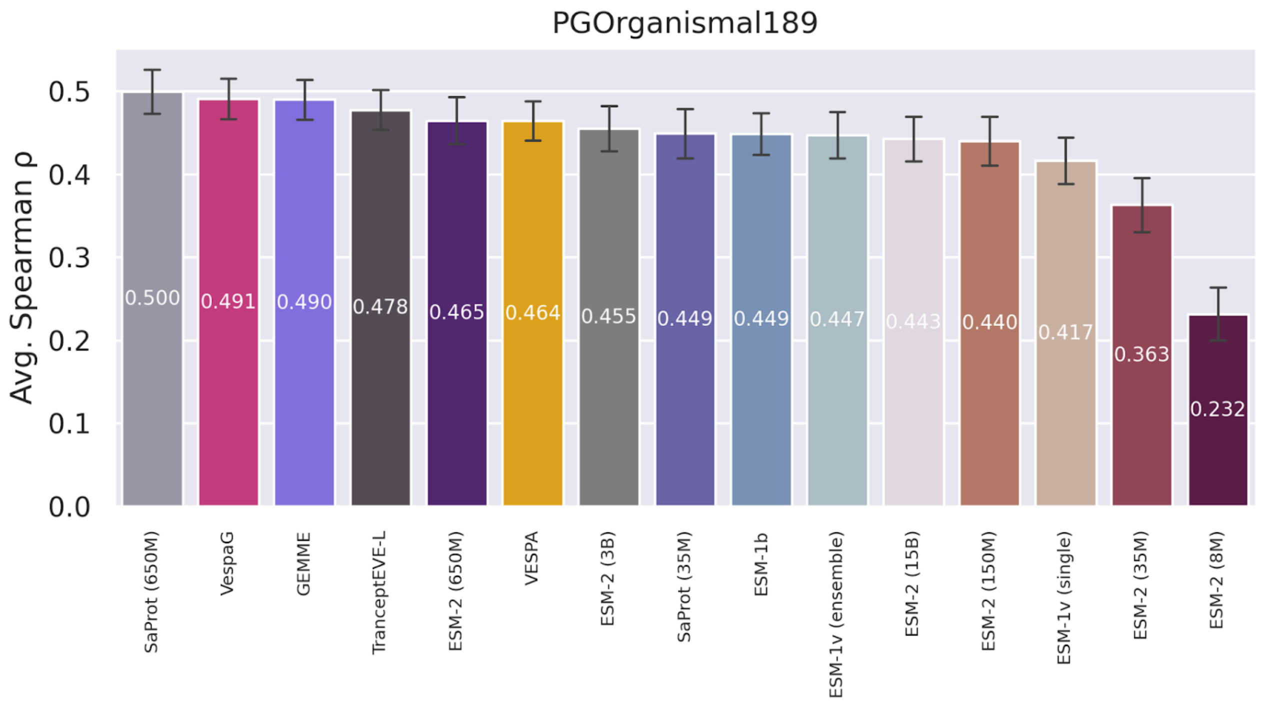

Figure S8: Average Spearman correlation coefficient ρ between experimental and predicted substitution effect scores of VespaG and SOTA methods on 189 organismal DMS assays of the ProteinGym substitution benchmark. Methods presented are VespaG, GEMME (Laine et al., 2019), TranceptEVE L (Notin et al., 2022), VESPA (Marquet et al., 2022), 6 variants of ESM-2 (Lin et al., 2023), 2 variants of SaProt (Su et al., 2024), 2 variants of ESM-1v (Meier et al., 2021), and ESM-1b (Rives et al., 2021). Results for all methods except VespaG, GEMME, and VESPA were downloaded from ProteinGym.

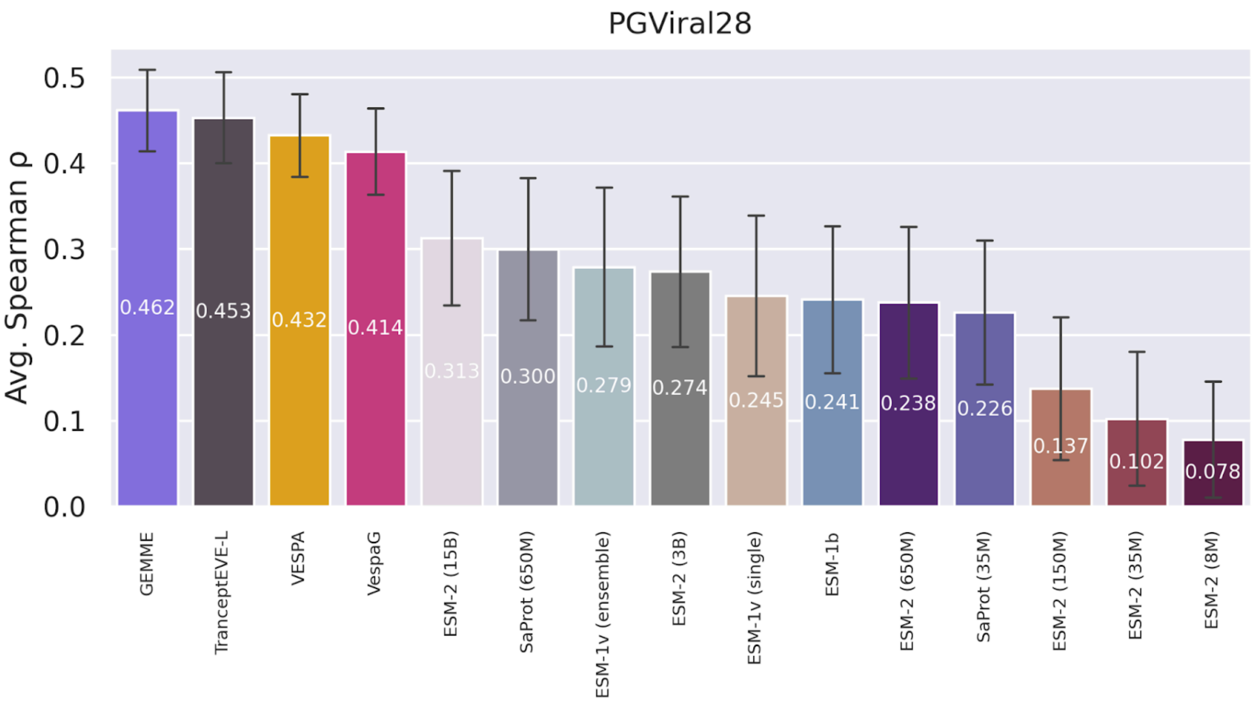

Figure S9: Average Spearman correlation coefficient ρ between experimental and predicted substitution effect scores of VespaG and SOTA methods on 28 viral DMS assays of the ProteinGym substitution benchmark. Methods presented are VespaG, GEMME (Laine et al., 2019), TranceptEVE L (Notin et al., 2022), VESPA (Marquet et al., 2022), 6 variants of ESM-2 (Lin et al., 2023), 2 variants of ESM-1v (Meier et al., 2021), and ESM-1b (Rives et al., 2021). Results for all methods except VespaG, GEMME, and VESPA were downloaded from ProteinGym.

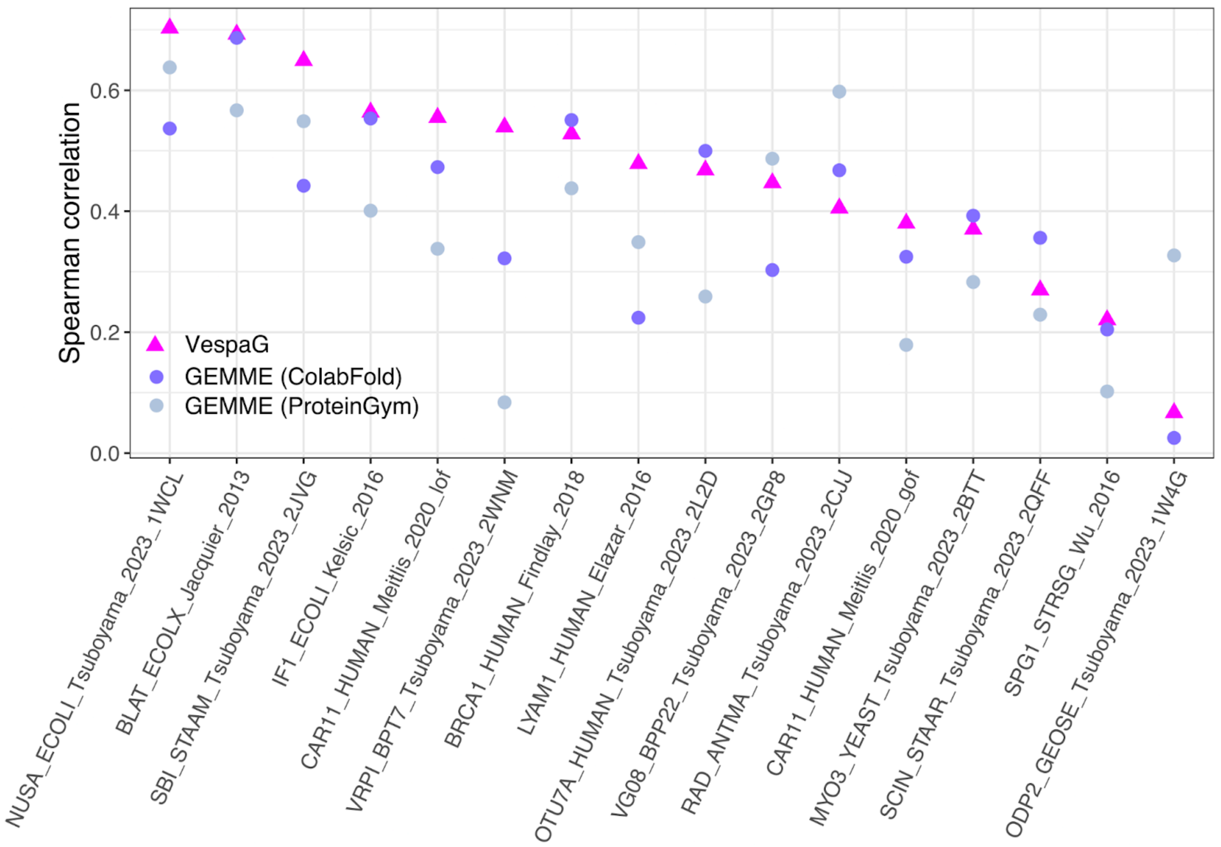

Figure S10: Comparison of Spearman correlation depending on the input alignment. We consider the subset of DMS where GEMME Spearman correlation varied by more than 0.1 between two different input alignment generation protocols, namely the MMseqs2-based strategy implemented in ColabFold (this work) (Laine et al., 2019; Mirdita et al., 2022) and the JackHMMER-based strategy (Johnson et al., 2010) implemented in ProteinGym.

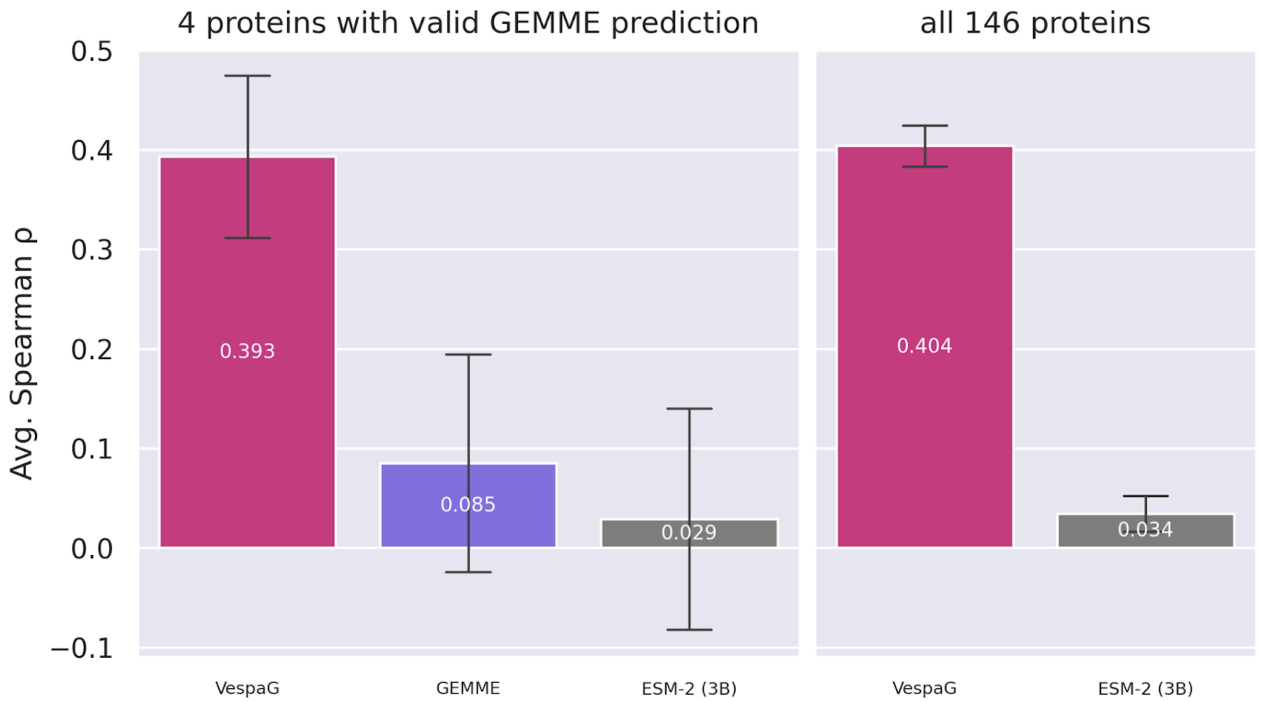

Figure S11: Average Spearman correlation coefficient ρ between predicted mutational effect scores and experimental ΔΔG scores of VespaG, GEMME (Laine et al., 2019), and ESM-2 (3B) (Lin et al., 2023) on the StabilityDeNovo146 dataset (Tsuboyama et al., 2023). The left subplot shows four proteins for which GEMME could produce predictions, and the right subplot excluding GEMME shows all 146 proteins.

REFERENCES

Brandes, N., Goldman, G., Wang, C. H., Ye, C. J. Ntranos, V.. 2023. “Genome-Wide Prediction of Disease Variant Effects with a Deep Protein Language Model.” *Nature Genetics* 55(9): 1512–22. doi:10.1038/s41588-023-01465-0.

Cheng, J., Novati, G., Pan, J., Bycroft, C., Žemgulytė, A., Applebaum, T., Pritzel, A., Wong, L.H., Zielinski, M., Sargeant, T., Schneider, R.G., Senior, A.W., Jumper, J., Hassabis, D., Kohli, P., Avsec, Ž., 2023. Accurate proteome-wide missense variant effect prediction with AlphaMissense. Science 381, eadg7492. https://doi.org/10.1126/science.adg7492

Elnaggar, A., Heinzinger, M., Dallago, C., Rehawi, G., Wang, Y., Jones, L., Gibbs, T., Feher, T., Angerer, C., Steinegger, M., Bhowmik, D., Rost, B., 2021. ProtTrans: Towards Cracking the Language of Life’s Code Through Self-Supervised Learning. https://doi.org/10.1101/2020.07.12.199554

Figliuzzi, M., Jacquier, H., Schug, A., Tenaillon, O., Weigt, M. 2016. “Coevolutionary Landscape Inference and the Context-Dependence of Mutations in Beta-Lactamase TEM-1.” *Molecular Biology and Evolution* 33(1): 268–80. doi:10.1093/molbev/msv211.

Frazer, J., Notin, P., Dias, M., Gomez, A., Min, J. K., Brock, K., Gal, Y., Marks, D. S. 2021. “Disease Variant Prediction with Deep Generative Models of Evolutionary Data.” *Nature* 599(7883): 91–95. doi:10.1038/s41586-021-04043-8.

Heinzinger, M., Weissenow, K., Gomez Sanchez, J., Henkel, A., Mirdita, M., Steinegger, M., Rost, B. 2024. “Bilingual Language Model for Protein Sequence and Structure.” : 2023.07.23.550085. doi:10.1101/2023.07.23.550085.

Hopf, T. A., Ingraham, J. B., Poelwijk, F. J., Schärfe, C. P. I., Springer, M., Sander, C., Marks, D. S. 2017. “Mutation Effects Predicted from Sequence Co-Variation.” *Nature Biotechnology* 35(2): 128–35. doi:10.1038/nbt.3769.

Johnson, L.S., Eddy, S.R., Portugaly, E., 2010. Hidden Markov model speed heuristic and iterative HMM search procedure. BMC Bioinformatics 11, 431. https://doi.org/10.1186/1471-2105-11-431

Laine, E., Karami, Y., Carbone, A., 2019. GEMME: A Simple and Fast Global Epistatic Model Predicting Mutational Effects. Mol. Biol. Evol. 36, 2604–2619. https://doi.org/10.1093/molbev/msz179

Lin, Z., Akin, H., Rao, R., Hie, B., Zhu, Z., Lu, W., Smetanin, N., Verkuil, R., Kabeli, O., Shmueli, Y., dos Santos Costa, A., Fazel-Zarandi, M., Sercu, T., Candido, S., Rives, A., 2023. Evolutionary-scale prediction of atomic-level protein structure with a language model. Science 379, 1123–1130. https://doi.org/10.1126/science.ade2574

Marquet, C., Heinzinger, M., Olenyi, T., Dallago, C., Erckert, K., Bernhofer, M., Nechaev, D., Rost, B., 2022. Embeddings from protein language models predict conservation and variant effects. Hum. Genet. 141, 1629–1647. https://doi.org/10.1007/s00439-021-02411-y

Meier, J., Rao, R., Verkuil, R., Liu, J., Sercu, T., Rives, A., 2021. Language models enable zero-shot prediction of the effects of mutations on protein function. https://doi.org/10.1101/2021.07.09.450648

Mirdita, M., Schütze, K., Moriwaki, Y., Heo, L., Ovchinnikov, S., Steinegger, M., 2022. ColabFold: Making Protein folding accessible to all. Nat. Methods. https://doi.org/10.1038/s41592-022-01488-1

Nijkamp, E., Ruffolo, J., Weinstein, E. N., Naik, N., Madani, A. 2022. “ProGen2: Exploring the Boundaries of Protein Language Models.” doi:10.48550/arXiv.2206.13517.

Notin, P., Dias, M., Frazer, J., Marchena-Hurtado, J., Gomez, A., Marks, D. S., Gal, Y. 2022. “Tranception: Protein Fitness Prediction with Autoregressive Transformers and Inference-Time Retrieval.” doi:10.48550/ARXIV.2205.13760.

Notin, P., Kollasch, A.W., Ritter, D., Niekerk, L. van, Paul, S., Spinner, H., Rollins, N., Shaw, A., Weitzman, R., Frazer, J., Dias, M., Franceschi, D., Orenbuch, R., Gal, Y., Marks, D.S., 2023. ProteinGym: Large-Scale Benchmarks for Protein Design and Fitness Prediction. https://doi.org/10.1101/2023.12.07.570727

Notin, P., Niekerk, L.V., Kollasch, A.W., Ritter, D., Gal, Y., Marks, D.S., 2022. TranceptEVE: Combining Family-specific and Family-agnostic Models of Protein Sequences for Improved Fitness Prediction. https://doi.org/10.1101/2022.12.07.519495

Rao, R. M., Liu, J., Verkuil, R., Meier, J., Canny, J., Abbeel, P., Sercu, T., Rives, A. 2021. “MSA Transformer.” In *Proceedings of the 38th International Conference on Machine Learning*, PMLR, 8844–56. https://proceedings.mlr.press/v139/rao21a.html (June 21, 2022).

Riesselman, A. J., Ingraham, J. B., Marks, D. S. 2018. “Deep Generative Models of Genetic Variation Capture the Effects of Mutations.” *Nature methods* 15(10): 816–22.

Rives, A., Meier, J., Sercu, T., Goyal, S., Lin, Z., Liu, J., Guo, D., Ott, M., Zitnick, C.L., Ma, J., Fergus, R., 2021. Biological structure and function emerge from scaling unsupervised learning to 250 million protein sequences. Proc. Natl. Acad. Sci. 118, e2016239118. https://doi.org/10.1073/pnas.2016239118

Su, J., Han, C., Zhou, Y., Shan, J., Zhou, X., Yuan, F., 2024. SaProt: Protein Language Modeling with Structure-aware Vocabulary. https://doi.org/10.1101/2023.10.01.560349

Tan, Y., Zhou, B., Zheng, L., Fan, G., Hong, L. 2024. “Semantical and Geometrical Protein Encoding Toward Enhanced Bioactivity and Thermostability.” : 2023.12.01.569522. doi:10.1101/2023.12.01.569522.

Truong Jr, T.F., Bepler, T., 2023. PoET: A generative model of protein families as sequences-of-sequences.

Tsuboyama, K., Dauparas, J., Chen, J., Laine, E., Mohseni Behbahani, Y., Weinstein, J.J., Mangan, N.M., Ovchinnikov, S., Rocklin, G.J., 2023. Mega-scale experimental analysis of protein folding stability in biology and design. Nature 620, 434–444. https://doi.org/10.1038/s41586-023-06328-6
